# pH-Responsive Polyethylene Glycol Engagers for Enhanced Brain Delivery of PEGylated Nanomedicine to Treat Glioblastoma

**DOI:** 10.1021/acsnano.4c05906

**Published:** 2025-01-03

**Authors:** Jun-Lun Meng, Zi-Xuan Dong, Yan-Ru Chen, Meng-Hsuan Lin, Yu-Ching Liu, Steve R. Roffler, Wen-Wei Lin, Chin-Yuan Chang, Shey-Cherng Tzou, Tian-Lu Cheng, Hsiao-Chen Huang, Zhi-Qin Li, Yen-Cheng Lin, Yu-Cheng Su

**Affiliations:** † Department of Biological Science and Technology, Center for Intelligent Drug Systems and Smart Bio-devices (IDS^2^B), National Yang Ming Chiao Tung University, Hsinchu 300, Taiwan; ‡ Institute of Biomedical Sciences, Academia Sinica, Taipei, 115, Taiwan; § Graduate Institute of Medicine, College of Medicine, Kaohsiung Medical University, Kaohsiung 807, Taiwan; ∥ School of Post-Baccalaureate Medicine, College of Medicine, Kaohsiung Medical University, Kaohsiung 807, Taiwan; ⊥ Department of Biomedical Science and Environmental Biology, Drug Development and Value Creation Research Center, Kaohsiung Medical University, Kaohsiung 807, Taiwan

**Keywords:** poly(ethylene glycol) (PEG), pH-responsive PEG engager, PEGylated nanomedicine, transferrin receptor (TfR), blood–brain barrier (BBB), glioblastoma (GBM)

## Abstract

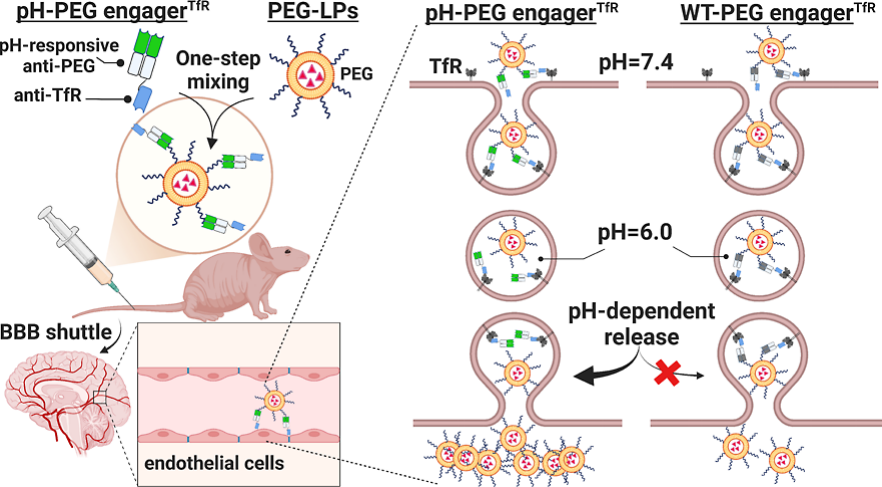

The blood–brain barrier (BBB) remains a major obstacle for effective delivery of therapeutics to treat central nervous system (CNS) disorders. Although transferrin receptor (TfR)-mediated transcytosis is widely employed for brain drug delivery, the inefficient release of therapeutic payload hinders their efficacy from crossing the BBB. Here, we developed a pH-responsive anti-polyethylene glycol (PEG) × anti-TfR bispecific antibody (pH-PEG engager^TfR^) that can complex with PEGylated nanomedicine at physiological pH to trigger TfR-mediated transcytosis in the brain microvascular endothelial cells, while rapidly dissociating from PEGylated nanomedicine at acidic endosomes for efficient release of PEGylated nanomedicine to cross the BBB. The pH-PEG engager^TfR^ significantly increased the accumulation of PEGylated nanomedicine in the mouse brain compared to wild-type PEG engager^TfR^ (WT-PEG engager^TfR^). pH-PEG engager^TfR^-decorated PEGylated liposomal doxorubicin exhibited an enhanced antitumor effect and extended survival in a human glioblastoma (GBM) orthotopic xenograft mice model. Conditional release of PEGylated nanomedicine during BBB-related receptor-mediated transcytosis by pH-PEG engager^TfR^ is promising for enhanced brain drug delivery to treat CNS disorders.

Central nervous system (CNS) disorders represent a wide range of brain-related diseases including brain infection, neurodegenerative diseases, lysosomal storage diseases (LSDs), and glioblastoma (GBM).^1,2^ Among these CNS disorders, GBM is one of the most aggressive and lethal disease. GBM is the common primary brain tumor with a poor prognosis, in which the median survival time for GBM patients is less than 2 years.^3^ The standard therapies for GBM include surgical resection combined with radiotherapy and chemotherapeutic drugs, such as temozolomide. However, temozolomide showed limited overall response rate ranging from 14 to 22.5% in GBM patients.^4,5^ Despite many chemotherapeutic drugs being developed to inhibit the growth of cancer cells, including GBM, the systemic delivery of chemotherapeutic drugs for treating GBM is a big challenge due to the blood–brain-barrier (BBB).^6^ The BBB is a specialized barrier formed by tight junctions between adjoining brain microvascular endothelial cells (BMECs) that limits the accessibility of small molecule drugs and macromolecules into the brain, while allowing penetration of essential nutrients via selective transporters.^7^ Therefore, developing efficient drug delivery systems for crossing the BBB is a major task for GBM therapies.

Various drug delivery strategies have been developed for crossing the BBB. For example, intracerebroventricular drug administration^8^ and convection-enhanced delivery^9^ can ensure direct drug administration into the brain. However, there are concerns that these invasive approaches may cause infection and brain damage.^10,11^ On the other hand, although the transient opening of BBB induced by focused ultrasound^12,13^ or vascular endothelial growth factor enhances drug delivery across the BBB,^14^ unwanted leakage of compounds and serum proteins into the brain might elevate the risk of neurotoxicity.^15^ Alternatively, receptor-mediated transcytosis is a promising approach to deliver therapeutics across the BBB in a safe manner.^16−18^

Current studies have shown that efficient cargo dissociation from BBB permeability-related receptors after receptor-mediated transcytosis is crucial for effective brain drug delivery. For example, fine-tuning anti-transferrin receptor (TfR) antibodies with lower affinity are more likely to dissociate from TfRs after transcytosis, leading to enhanced uptake of TfR antibodies in the brain.^19,20^ Similarly, pH-sensitive anti-TfR antibodies exhibited improved brain delivery due to conditional dissociation of anti-TfR antibodies from TfRs in the acidic endosomes during transcytosis.^21^ However, therapeutics must be linked to these engineered TfR-targeting moieties as fusion protein modalities, which heavily limits their flexibility in choosing BBB-shuttle ligands and appropriate drugs.

Nanomedicine is able to encapsulate a large amount of drugs and diminish drug leakage to avoid unwanted toxicity.^22,23^ The accumulation of nanomedicine in tumors relies on the enhanced permeability and retention effect.^24,25^ However, the enhanced permeability and retention effect is heterogeneous in different cancer types, thereby limiting the therapeutic efficacy of nanomedicine.^26,27^ Functionalization of nanomedicine with targeting ligands significantly improves cancer-specific delivery and cellular uptake of nanomedicine.^28,29^ Poly(ethylene glycol) (PEG) modification of nanomedicine is commonly used to prevent their clearance by the reticuloendothelial system.^30−32^ Therefore, we have successfully developed bispecific PEG engager systems for tumor-specific delivery of PEGylated nanomedicine to treat cancers.^28,33−35^

To further boost brain uptake of PEGylated nanomedicine, here, we engineered a pH-responsive anti-PEG × anti-TfR bispecific antibody (pH-PEG engager^TfR^) platform, in which the pH-responsive anti-PEG arm can bind to PEG in a pH-dependent manner. The pH-PEG engager^TfR^ can noncovalently couple with PEGylated nanomedicine at physiological pH, followed by dissociation of PEGylated nanomedicine in acidified endosomes (pH 6.0) after TfR-mediated transcytosis to efficiently cross the BBB (Figure 1). Structure-guided engineering of pH-responsive anti-PEG Fabs was performed to generate pH-PEG engagers. We further examined the PEG-binding activity of the pH-PEG engagers under different pH conditions. We then investigated whether pH-PEG engager^TfR^ can facilitate PEGylated nanomedicine to traverse the BBB in vitro. Finally, the enhanced brain accumulation and therapeutic efficacy of pH-PEG engager^TfR^-decorated PEGylated nanomedicine were determined in an orthotopic human glioblastoma (GBM) xenograft mice model. In summary, this generic pH-PEG engager might be a simple and flexible platform to efficiently deliver various PEGylated therapeutics across the BBB to treat CNS-related disorders.

**Figure 1:**
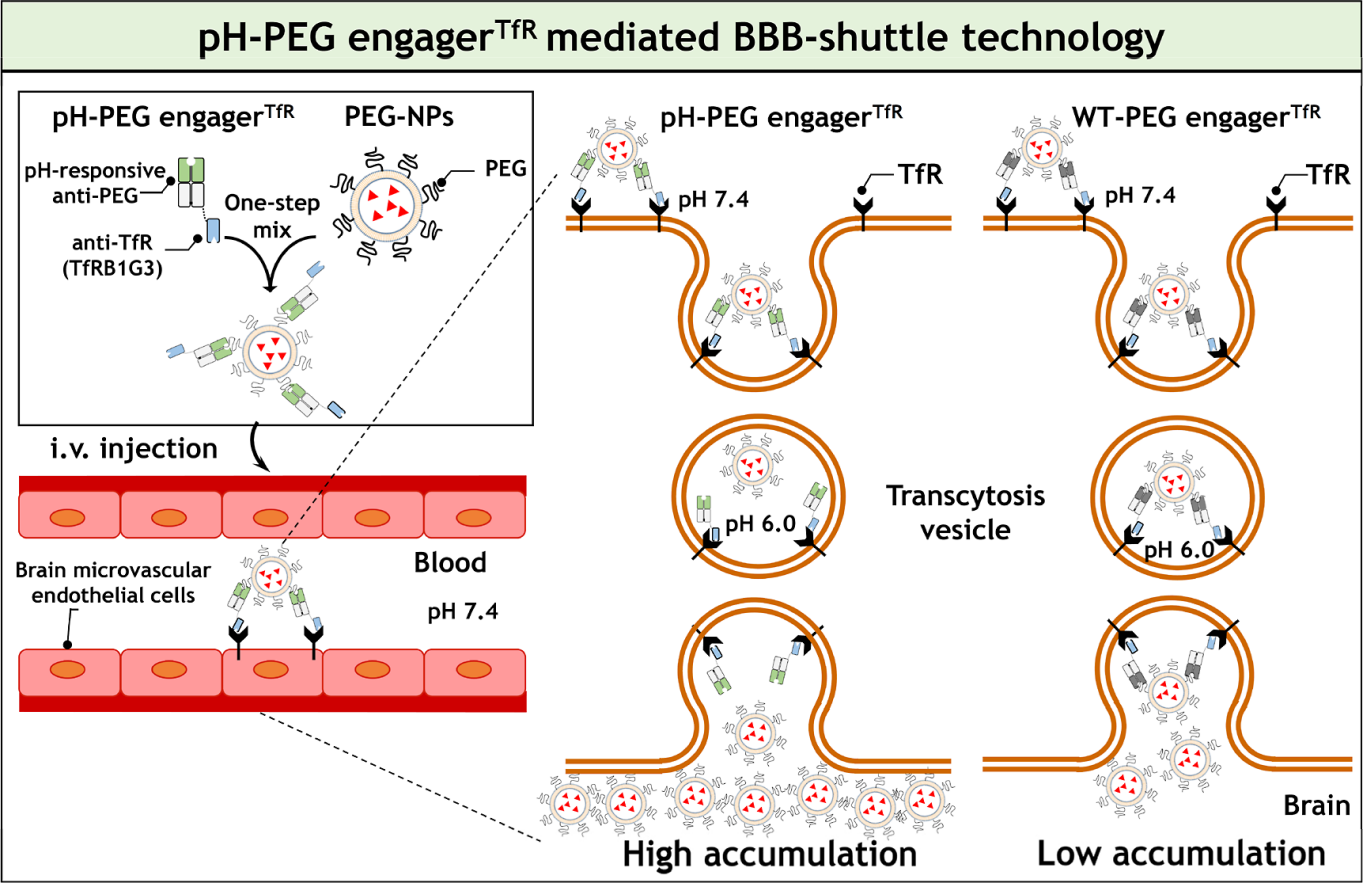
Overview of pH-PEG engager^TfR^-mediated BBB-shuttle nanomedicine delivery strategy. Wild-type PEG engager^TfR^ (WT-PEG engager^TfR^) and pH-PEG engager^TfR^ are generated by fusing parental anti-PEG Fab or pH-responsive anti-PEG Fab with a TfR-binding domain (TfRB1G3). TfR-targeting PEGylated liposomes (PEG-LPs) can be simply prepared by one-step mixing of pH-PEG engager^TfR^ with PEG-LPs and subsequently trigger TfR-mediated transcytosis in BMECs. The dissociation of pH-PEG engager^TfR^-decorated PEG-LPs in acidic endosomes facilitates greater accumulation of PEG-LPs across the BBB as compared to WT-PEG engager^TfR^-decorated PEG-LPs.

## Results

### Structure-Guided Design of pH-Responsive PEG Engagers

**Figure 2:**
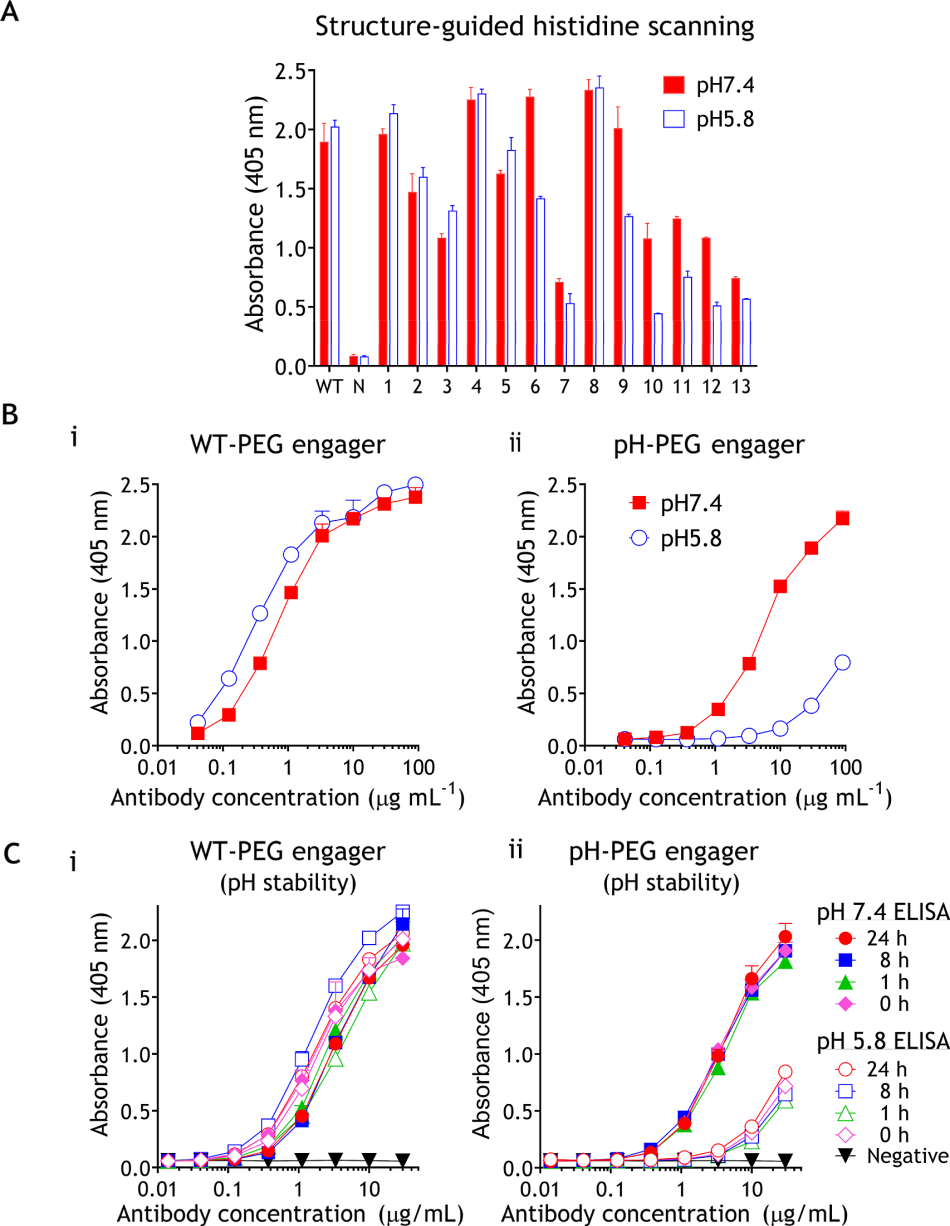
Screening of pH-responsive PEG engagers. Microplate wells coated with amino-PEG-LPs were incubated with PEG engager variants (A), graded concentrations of WT-PEG engager, or pH-PEG engager (B). After 1 h, the wells were washed at different pH conditions (pH 7.4 or pH 5.8), and antibody binding was determined by incubating HRP-conjugated goat anti-human F(ab’)_2_ fragment-specific antibodies, followed by the ABTS substrate. (C) pH-PEG engagers were incubated at pH 5.8 for 1 h or 24 h and then diluted into phosphate-buffered saline (PBS) (pH 7.4). Graded concentrations of acid-treated pH-PEG engagers were added to the microplates coated with amino-PEG-LPs for determining their PEG-binding activity at different pH values (pH 7.4 and pH 5.8) by using anti-PEG ELISA as shown above. The WT-PEG engager (WT) and DNS engager (N) were used as positive and negative control groups, respectively. The results show the mean absorbance values (405 nm) ± standard deviation (*n* = 3).

To engineer the pH-responsive PEG engagers, we determined the cocrystal structure of humanized anti-PEG 6.3 Fab (h6.3) in complex with PEG. The h6.3 Fab/PEG cocrystal (Protein Data bank entry 8Z95) revealed similar structures with the mouse-human chimeric 6.3 Fab/PEG cocrystal,^36^ in which h6.3 Fab also formed homodimers, while interacting with PEG (Figure S1 and Table S1). We hypothesized that the conditional disruption of the formation of the h6.3 Fab homodimer/PEG complex under low pH conditions may decrease its PEG-binding activity in a pH-dependent manner. The pH-responsive protein interactions typically rely on the presence of ionizable amino acids at the binding interface. The protonation of ionizable amino acids at low pH conditions could induce intramolecular electrostatic attraction, repulsion, or hydrogen bond formation, leading to decreased or increased binding activity at different pH conditions.^37−39^ Therefore, we performed a structure-guided mutagenesis of the residues corresponding to h6.3 dimerization using ionizable amino acid substitution (histidine or glutamic acid) to generate pH-responsive anti-PEG antibodies. The crude protein of h6.3 mutants expressed in *Escherichia coli* C43 (DE3) was extracted and screened by an anti-PEG enzyme-linked immunosorbent assay (ELISA) at different pH conditions. Several h6.3 Fab variants displayed pH-dependent PEG-binding activity (Figure 2A). Finally, the h6.3 Fab variants that produced an absorbance reading higher than 1.0 unit at pH 7.4, while decreasing more than 25% in PEG-binding activity at pH 5.8 (Clone 6, 9, 10, 11, and 12), were combined to generate the pH-PEG engager. To determine the pH-dependent PEG-binding of the pH-PEG engager, we analyzed the pH-PEG engager at different pH values using ELISA and included the pH-insensitive wild-type PEG engager (WT-PEG engager) as a control. Figure 2B shows that the WT-PEG engager strongly bound to PEG-coated ELISA plates at both pH 7.4 and pH 5.8 conditions. By contrast, the pH-PEG engager could strongly bind to PEG-coated plates at pH 7.4, while its PEG-binding activity decreased at pH 5.8, demonstrating that the pH-PEG engager exhibits pH-responsive PEG-binding activity (Figure 2B). To investigate the pH stability of pH-PEG engagers, these antibodies were treated with an acidic buffer (pH 5.8) for different incubation times ranging from 1 to 24 h, followed by analyzing their PEG-binding activity at different pH values (pH 7.4 and pH 5.8) by ELISA. Figure 2C shows that long-term acidic treatment did not impair the PEG-binding activity of PEG engager variants, indicating that the pH-PEG engager displays reversible pH-sensitive PEG-binding activity and long-term pH stability.

### Production and Analysis of Bispecific pH-PEG Engager^TfR^

**Figure 3:**
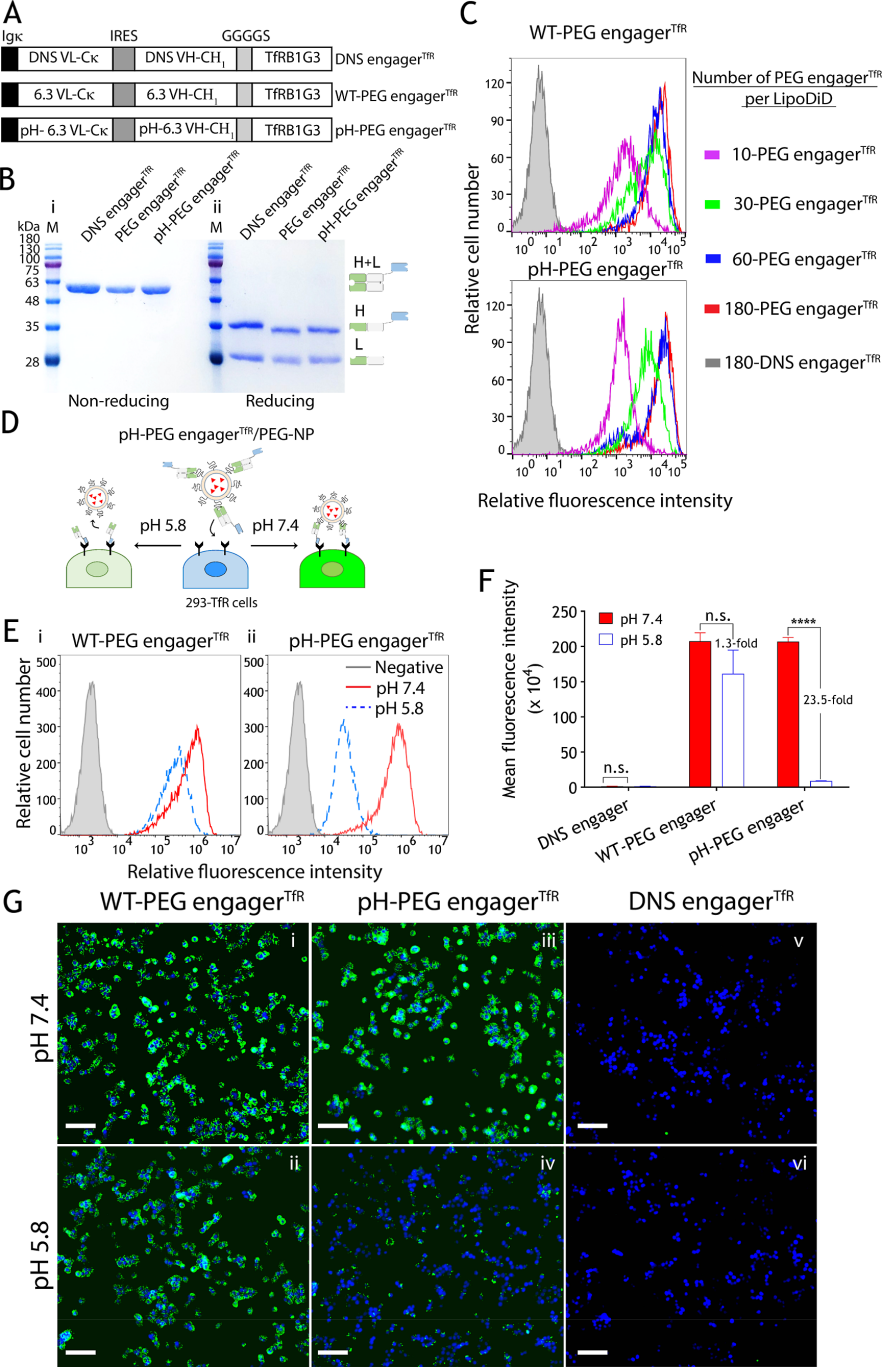
Characterization of pH-responsive PEG engager^TfR^. (A) Schematic of DNS engager^TfR^, WT-PEG engager^TfR^, or pH-PEG engager^TfR^ constructs, which is composed of a murine Ig kappa chain leader sequence (Igκ), an antibody light chain (LC) (VL-Cκ of DNS, h6.3 or pH-responsive h6.3), an internal ribosome entry site (IRES) sequence, a heavy chain (HC) (VH–CH_1_ of DNS, h6.3 or pH-responsive h6.3), a flexible linker peptide (GGGGS), an anti-transferrin receptor fragment (TfRB1G3), and a polyhistidine-tag (His tag). (B) SDS-PAGE analysis under reduced (i) and nonreduced (ii) conditions showing Coomassie Blue staining of purified DNS engager^TfR^, PEG engager^TfR^, and pH-PEG engager^TfR^. M, PageRuler prestained protein ladder (Fermentas). HC (heavy chain). LC (light chain). (C) Live 293-mTfR cells were immunofluorescence stained with various coupling ratios of PEG engager^TfR^-decorated fluorescent PEG-lipoDiD or control DNS engager^TfR^-decorated fluorescent PEG-lipoDiD and then analyzed on a flow cytometer. (D) Schematic outline of pH-responsive PEG-binding activity of pH-PEG engager^TfR^-decorated PEG-liposomes targeting TfR-positive cells. (E) Live 293-mTfR cells were immunofluorescence stained with control DNS engager^TfR^ (gray area) or PEG engager^TfR^ (i) or pH-PEG engager^TfR^ (ii)-decorated fluorescent PEG-lipoDiD at pH 7.4 (red solid line) or pH 5.8 (blue dashed line) conditions and then analyzed on a flow cytometer. (F) The uptake of PEG-lipoDiD in 293-TfR cells at different pH conditions was determined by measuring mean fluorescence intensities (*n* = 3). Data are shown as mean ± s.d. Significant differences in mean fluorescent intensity between pH7.4 and pH5.8 conditions are indicated: ****P ≤ 0.0001 (two-way analysis of variance (ANOVA)). n.s., not significant. (G) PEG engager^TfR^ (i,ii), pH-PEG engager^TfR^ (iii,iv) and DNS engager^TfR^ (v,vi)-decorated PEG-liopoDiO (green) supplemented with Hoechst 33342 (blue) were incubated with 293-TfR cells under pH 7.4 (i,iii,v) or pH 5.8 (ii,iv,vi) conditions and then observed with a digital fluorescence microscope. Scale bars, 25 μm. Representative images from three independent experiments are shown.

The humanized wild-type (WT) anti-PEG h6.3 Fab,^28^ pH-responsive anti-PEG h6.3 Fab, or anti-dansyl (DNS) Fab^40^ fragments were genetically fused to an anti-transferrin receptor (TfR) domain (TfRB1G3) with a similar binding pattern to mouse TfR and human TfR (Figure S2)^41^ via a flexible GGGGS peptide linker to generate bispecific WT-PEG engager^TfR^, pH-PEG engager^TfR^, and control DNS engager^TfR^ (Figure 3A). The DNS engager^TfR^, WT-PEG engager^TfR^, and pH-PEG engager^TfR^ were produced by using an ExpiCHO mammalian expression system. The purified DNS engager^TfR^, WT-PEG engager^TfR^, and pH-PEG engager^TfR^ were analyzed by 12.5% sodium dodecyl sulfate polyacrylamide gel electrophoresis (SDS–PAGE) displaying the expected molecular weights of intact bispecific antibodies that composed a 55 kDa fragment under nonreducing conditions and a 31 kDa heavy chain fragment and a 24 kDa light chain fragment under reducing conditions (Figure 3B).

To determine the optimal predocking ratio of PEG engagers and PEG-LPs for the preparation of stable TfR-targeting liposomes, DNS engager^TfR^, WT-PEG engager^TfR^, or pH-PEG engager^TfR^ was mixed with PEG-LPs at different ratios, resulting in 10, 30, 60, and 180 PEG engager^TfR^ (WT-PEG engager^TfR^ or pH-PEG engager^TfR^) per liposome. We verified that the conjugation rates of WT-PEG engager^TfR^ and pH-PEG engager^TfR^ on PEG-LPs were greater than 98% (Figure S3). We compared the TfR-binding activity of the PEG engager^TfR^-decorated fluorescent PEG-liposomal DiD (PEG-lipoDiD) to HEK293 cells that overexpressed mouse TfR (293-mTfR) and analyzed it by a flow cytometer. Figure 3C shows that the fluorescent intensity of PEG engager^TfR^-decorated PEG-lipoDiD on 293-mTfR cells was gradually increased as the density of PEG engager^TfR^ on PEG-lipoDiD increased. The formation of complexes between PEG engager^TfR^ and PEG-LPs at different ratios was examined by measuring the average size of PEG-LPs with or without PEG engager complexation using dynamic light scattering (Figures S4–S7). The 10-PEG engager^TfR^-LP groups were not included due to their weak TfR-binding activity (Figure 3C). The particle size of PEG engager^TfR^-LPs was increased as compared to PEG-LPs alone (Figures S4–S7 and S8E, PEG engager^TfR^-LPs: 97–103 nm versus PEG-LPs: 94–95 nm, *P* < 0.001) or DNS engager^TfR^-LPs (Figures S4–S7 and S8E, PEG engager^TfR^-LPs: 97–103 nm versus DNS engager^TfR^-LPs: 93–95 nm, *P* < 0.001), suggesting that PEG engager^TfR^ but not DNS engager^TfR^ can form a complex with PEG-LPs and Doxisome (Figures S4–S7). The zeta potential of these PEG engager^TfR^-decorated PEG-LPs was slightly more negative as compared to PEG-LPs alone or DNS engager^TfR^-LPs (PEG engager^TfR^-LPs: −11 to −19.4 mV, PEG-LPs and DNS engager^TfR^-LPs: −4.7 to −10.3 mV) (Figures S4–S7). In addition, 180-PEG engager^TfR^-LPs initiated particle aggregation (Figures S4–S7), while 30 and 60-PEG engager^TfR^-LPs remained nonaggregated and maintained their TfR-binding activity for 3 days after complexation (Figure S8). Taken together, 60-PEG engager^TfR^-lipoDiD and 180-PEG engager^TfR^-lipoDiD displayed similar TfR-binding activity (Figure 3C) but 180-PEG engager^TfR^-LPs were prone to aggregation (Figure S4). Therefore, we chose 60-PEG engager^TfR^-LPs to investigate whether the pH-PEG engager^TfR^-decorated PEG-LPs are dissociable in an acidic environment. The 293-mTfR cells were incubated with WT-PEG engager^TfR^, pH-PEG engager^TfR^, or control DNS engager^TfR^-decorated PEG-lipoDiD under pH 7.4 or 5.8 conditions at 4 °C, at which temperature transcytosis is inhibited. Unbound compounds were removed by washing with cold PBS and then analyzed by a flow cytometer and fluorescence microscopy (Figure 3D). Figure 3E(i) shows that compared to DNS engager^TfR^ negative control, WT-PEG engager^TfR^-decorated PEG-lipoDiD revealed similar binding activity at both pH 7.4 and pH 5.8 conditions. By contrast, the binding activity of pH-PEG engager^TfR^-decorated PEG-lipoDiD to 293-mTfR cells at pH 5.8 was decreased by 23.5-fold as compared with their binding activity at pH 7.4 [Figure 3E(ii),F]. Similarly, fluorescence microscope imaging showed that WT-PEG engager^TfR^-decorated PEG-lipoDiO strongly bound to 293-mTfR cells at both pH 7.4 and pH 5.8 [Figure 3G(i,ii)], whereas the fluorescent intensity of pH-PEG engager^TfR^-decorated PEG-lipoDiO in 293-TfR cells was preferentially accumulated at pH 7.4 but substantially reduced at the pH 5.8 condition [Figure 3G(iii,iv)]. By contrast, no binding of PEG-lipoDiO was detected in the DNS engager^TfR^ negative control group [Figure 3G(v,vi)]. These results suggested that pH-PEG engager^TfR^ could couple with PEG-LPs at neutral pH conditions for specific delivery of PEG-LPs to TfR-expressing cells but undergo efficient dissociation from decorated PEG-LPs at acidic pH conditions.

### pHPEG engager^TfR^ Facilitates PEG-LPs to Traverse the BBB In Vitro

**Figure 4:**
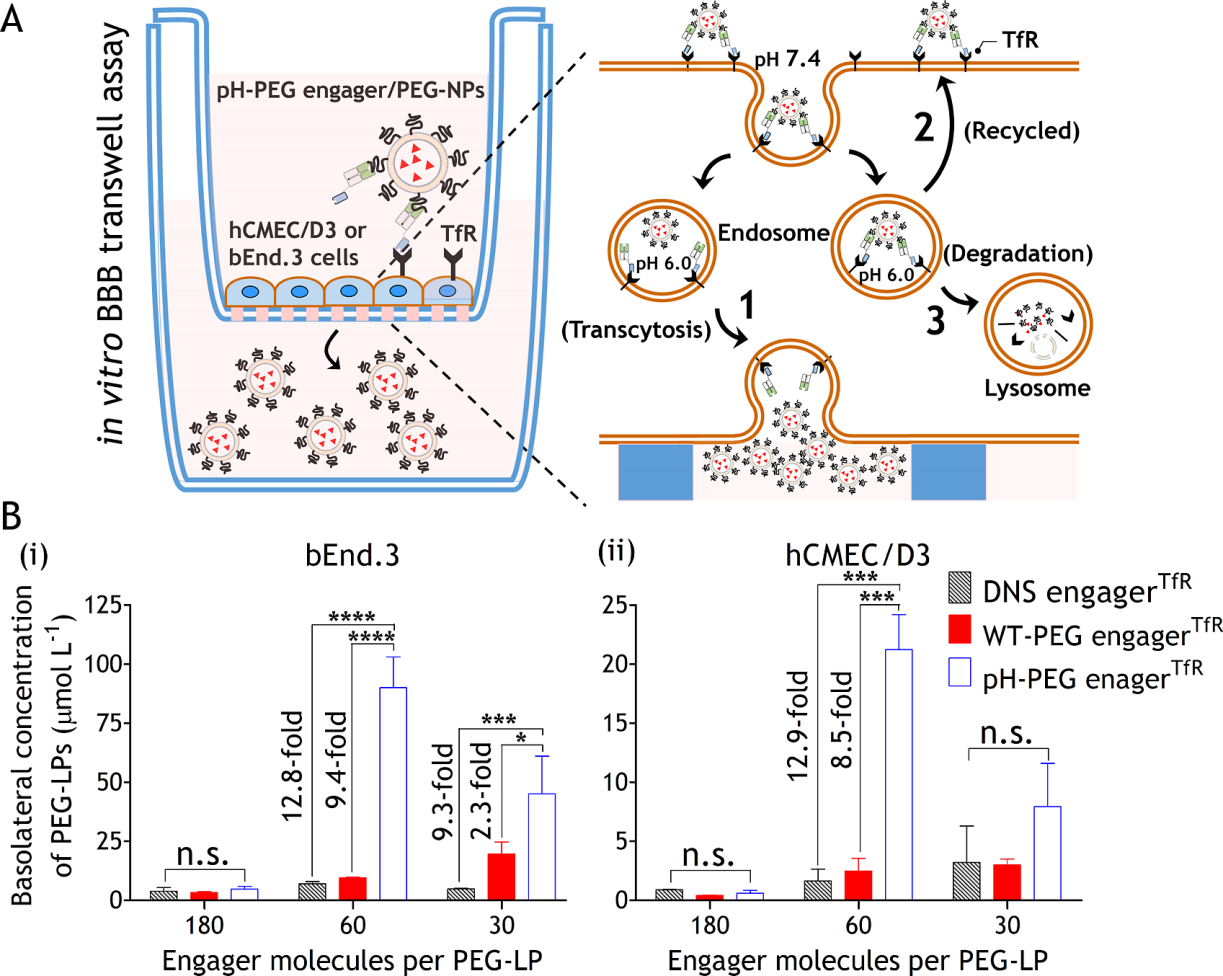
BBB transcytosis efficacy of pH-PEG engager^TfR^-decorated PEG-NPs. (A) Illustration of a pulse-chase in vitro BBB model established using bEnd.3 or hCMEC/D3 cells that show three potential endocytic sorting routes of pH-PEG engager^TfR^-decorated PEG-LPs. pH-PEG engager^TfR^-decorated PEG-LPs trigger TfR-mediated endocytosis that may be (1) rapidly dissociated at acidic endosomes for efficient transcytosis across the endothelial cells, (2) recycled back to the luminal side, or (3) transported to lysosomes for degradation. (B) The apical-to-basolateral delivery of DNS engager^TfR^- or PEG engager^TfR^-, or pH-PEG engager^TfR^-decorated PEG-LPs in bEnd.3 (i) or hCMEC/D3 (ii). BBB models were measured by using quantitative anti-PEG sandwich ELISAs. Two-way ANOVA was used for the statistical analysis. Data are shown as mean ± standard deviation. Significant differences in PEG-LP concentrations between treatment and control groups are indicated: *, *p* ≤ 0.0237, **, *p* ≤ 0.0077, ***, *p* ≤ 0.001, ****, *p* ≤ 0.0001.

To examine whether pH-PEG engager^TfR^ can facilitate PEG-LPs to traverse the BBB in vitro, we performed a pulse-chase transwell assay to prevent the paracellular flux effect, resulting from the leakage of mouse bEnd.3 and human hCMEC/D3 BMEC lines (Figure 4A).^42^ The bispecific antibodies, including DNS engager^TfR^, WT-PEG engager^TfR^, or pH-PEG engager^TfR^, were premixed with PEG-LPs at different antibody-to-DSPE-mPEG_2000_ molar ratios of 3:330, 6:330, and 18:330 at 4 °C for 1 h to produce 30, 60, and 180-engager^TfR^-LPs, respectively. The polycarbonate 24-well transwell membranes seeded with a monolayer of bEnd.3 or hCMEC/D3 were pulsed with DNS engager^TfR^-, WT-PEG engager^TfR^-, or pH-PEG engager^TfR^-decorated PEG-LPs for 1 h. For the chase phase, both the upper and lower compartments of transwell plates were washed and then incubated in culture medium for 2 h. The apical-to-basolateral transport of PEG-LPs was measured using quantitative anti-PEG sandwich ELISAs.^43−45^Figure 4B(i) shows that the degree of PEG-LP transport across the bEnd.3 cells was limited using 30 and 180-engager^TfR^-LPs. When using the engager/PEG-LP ratio of 60-engager^TfR^-LPs, pH-PEG engager^TfR^ exhibited 12.8-fold and 9.4-fold enhanced transcellular transport of PEG-LPs across the mouse BMEC line (bEnd.3) as compared to the DNS engager^TfR^ negative control and WT-PEG engager^TfR^, respectively. Likewise, 60-pH-PEG engager^TfR^-LPs also significantly facilitated PEG-LPs to traverse the hCMEC/D3 cells about 12.9-fold more efficiently than DNS engager^TfR^ negative control and 8.5-fold greater than WT-PEG engager^TfR^, whereas the rate of 30 and 180-engager^TfR^-LPs transport across the hCMEC/D3 was limited [Figure 4B(ii)]. The results indicated that 60-pH-PEG engager^TfR^-LP is the optimal formulation to traverse both the mouse and human BBB in vitro. This may be because the weak TfR-binding activity of 30-pH-PEG engager^TfR^-LPs (Figure 3C), which results in inefficient TfR-mediated transcytosis^46^ and the particle aggregation of 180-pH-PEG engager^TfR^-LPs (Figures S4–S7), which impairs receptor-mediated endocytosis.^47^ To further verify whether 60-pH-PEG engager^TfR^-LPs allow conditional release of PEG-LPs in acidic endosome (pH 6.0), hTfR-GFP overexpressing 293 cells were incubated with WT-PEG engager^TfR^-lipoDiD or pH-PEG engager^TfR^-lipoDiD (60 engager^TfR^ per PEG-lipoDiD) and then examined under a confocal microscope. Figure S9 shows that WT-PEG engager^TfR^-lipoDiD colocalized with TfR-GFP on the endosomal membranes (Figure S9A), while the PEG-lipoDiD was dissociated from the TfR-GFP/pH-PEG engager^TfR^ complex in acidic endosomes (Figure S9B). In summary, these results suggested that pH-PEG engager^TfR^ might rapidly dissociate with PEG-LPs at acidic endosomes after TfR-mediated transcytosis, leading to efficient cargo transport across the bEnd.3 and hCMEC/D3 endothelial cells.

### Enhanced Brain Delivery of PEG-LPs by the pH-PEG Engager^TfR^

**Figure 5:**
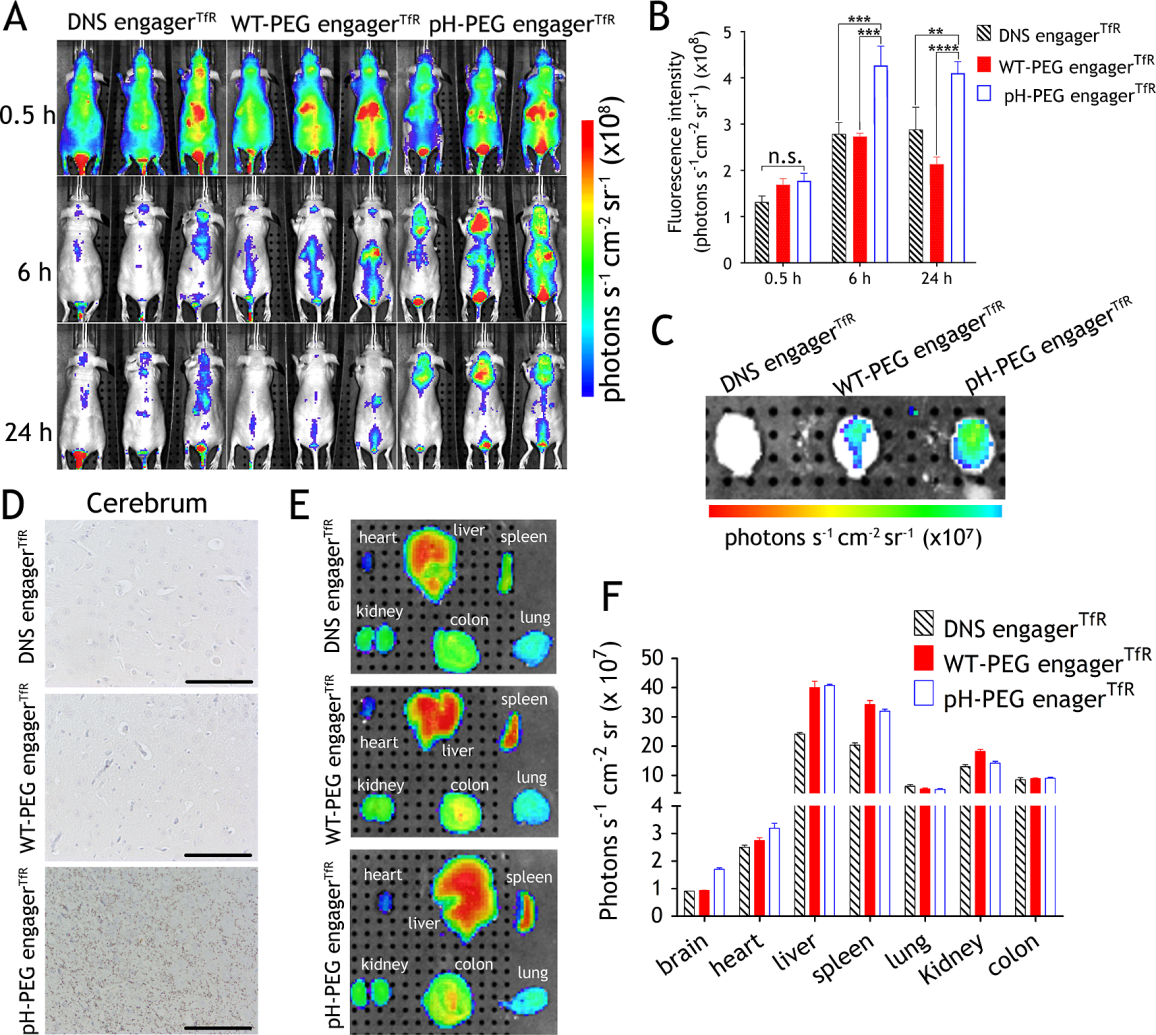
pH-PEG engager^TfR^ enhances brain uptake of PEG-LPs. (A) BALB/c nude mice were intravenously injected with WT-PEG engager^TfR^-, pH-PEG engager^TfR^-, or control DNS engager^TfR^-decorated PEG-lipoDiR and the whole-body imaging was imaged at 0.5 h, 6, and 24 h with an IVIS Spectrum imaging system (*n* = 3 mice). (B) The uptake of PEG-lipoDiR in mouse brains was determined by measuring fluorescence intensities (*n* = 3). Data are shown as mean ± standard deviation. Significant differences in mean fluorescent intensity between DNS engager^TfR^, WT-PEG engager^TfR^, and pH-PEG engager^TfR^ groups are indicated: **, *p* = 0.0011, ***, *p* ≤ 0.0002, ****, *p* ≤ 0.0001 (two-way ANOVA). n.s., not significant. (C) Ex vivo fluorescence imaging of PBS-perfused brains dissected from mice at 24 h postinjection. (D) PBS-perfused brain sections (cerebrum) collected from DNS or PEG engager-decorated PEG-lipoDiR-treated mice were stained with anti-PEG IgM for the detection of PEG-lipoDiR and followed by hematoxylin and eosin staining. Scale bars, 100 μm. (E) Ex vivo fluorescence imaging of PBS-perfused organs isolated from mice at 24 h postinjection. (F) Quantitative biodistribution of PEG-lipoDiR fluorescence intensity in PBS-perfused organs collected from DNS or PEG engager-decorated PEG-lipoDiR-treated mice at 24 h postinjection. Data are shown as mean ± standard deviation.

To determine whether pH-PEG engager^TfR^ can enhance the delivery of PEGylated nanomedicine across the BBB, BALB/c nude mice or intracranial GBM-bearing BALB/c nude mice were intravenously injected with WT-PEG engager^TfR^-, pH-PEG engager^TfR^-, or control DNS engager^TfR^-decorated fluorescent PEG-liposomal DiR (PEG-lipoDiR) using a dose based on the lipid amount of the therapeutic dose of PEGylated liposomal doxorubicin (Doxisome) (3 mg kg^–1^ of doxorubicin in Doxisome contains 500 nmole lipids per 20 g mouse). DNS engager^TfR^, WT-PEG engager^TfR^, or pH-PEG engager^TfR^ was premixed with PEG-lipoDiR to generate 60-engager^TfR^-lipoDiR. These mice were analyzed on an in vivo imaging system (IVIS) at 0.5, 6, and 24 h after injection. The fluorescent signals of these mice at 0.5 h after injection revealed no differences in the dose of engager^TfR^-lipoDiR administration between the groups (Figure 5A,B). The enhanced fluorescence signal was detected in the mouse brain treated with pH-PEG engager^TfR^-decorated PEG-lipoDiR as compared to the DNS engager^TfR^ and WT-PEG engager^TfR^ control groups at 6 and 24 h after injection (Figure 5A). The brain fluorescence intensity in pH-PEG engager^TfR^-decorated PEG-lipoDiR treated mice at 6 and 24 h was 1.56-fold and 1.9-fold higher than in the WT-PEG engager^TfR^ control group, respectively (Figure 5B). By contrast, WT-PEG engager^TfR^-decorated PEG-lipoDiR-treated mice exhibited similar brain fluorescence intensity as mice treated with DNS engager^TfR^ negative control (Figure 5B). Ex vivo imaging of isolated brain organs revealed that pH-PEG engager^TfR^ significantly enhanced the accumulation of PEG-lipoDiR in the mouse brain compared to DNS engager^TfR^ and WT-PEG engager^TfR^ control groups (Figure 5C). Additionally, high PEG-lipoDiR accumulation was homogeneously distributed in the brain sections from pH-PEG engager^TfR^-lipoDiR-treated mice after PBS perfusion, whereas in DNS engager^TfR^ and WT-PEG engager^TfR^ control groups, PEG-lipoDiR accumulation in the brain sections was limited. These results demonstrated that pH-PEG engager^TfR^-lipoDiR was localized in the brain tissues but not in the brain vasculature (Figure 5D). A biodistribution study was also performed in mice for tracking the accumulation of PEG-lipoDiR in mice. Compared to nontargeted control (DNS engager^TfR^-lipoDiR), the accumulation of TfR-targeted lipoDiR (WT-PEG engager^TfR^-lipoDiR or pH-PEG engager^TfR^-lipoDiR) was increased in liver and spleen (Figure 5E,F). The increased accumulation of TfR-targeted lipoDiR in liver and spleen might be due to the high TfR expression levels in liver and spleen.^48^ Likewise, the enhanced brain delivery of pH-PEG engager^TfR^-decorated PEG-lipoDiR in intracranial GBM-bearing BALB/c nude mice is consistent with nontumor mice groups (Figure S10). Furthermore, we tested the pharmacokinetics of PEG engagers docking with or without PEG-LPs to investigate whether predocking of PEG engagers with PEG-LPs could enhance their serum half-lives. The half-lives of the PEG engagers in PEG engager alone groups (WT-PEG engager^TfR^: 0.4 h and pH-PEG engager^TfR^: 0.43 h) were shorter than that of PEG engager-decorated PEG-LP groups (WT-PEG engager^TfR^-PEG-LP: 1.51 h and pH-PEG engager^TfR^-PEG-LP: 1.43 h), indicating that the intact predocked PEG-LPs sustain in the body over a prolonged period (Figure S11A–C). However, the half-lives of PEG-LPs (PEG-LP: 6.25 h, DNS engager^TfR^-PEG-LP: 6.47 h, WT-PEG engager^TfR^-PEG-LP: 6.53 h and pH-PEG engager^TfR^-PEG-LP: 6.3 h) were longer than those of PEG engager (1.43–1.51 h) in PEG engager-decorated PEG-LP groups, suggesting that the PEG engagers might gradually dissociate from PEG-LPs and be relatively unstable in vivo (Figure S11D). Therefore, we conclude that pH-PEG engager^TfR^ can efficiently deliver PEG-LPs across the mouse BBB, and multiple treatments should be used for anti-GBM therapy.

### Anti-GBM Efficacy of pH-PEG engager^TfR^-Decorated Doxisome on an Orthotopic Brain Tumor Model

**Figure 6:**
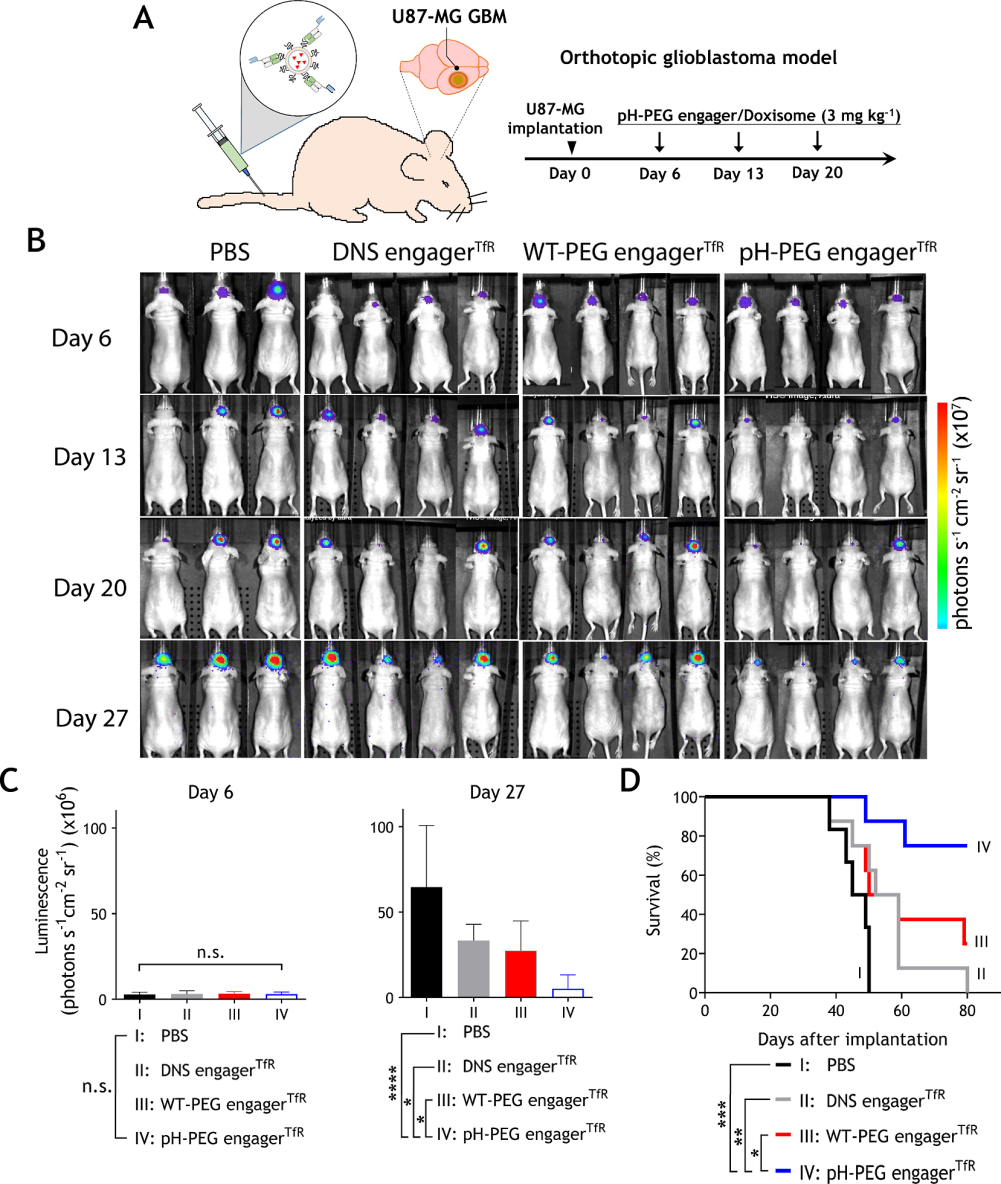
In vivo anti-GBM efficacy of pH-PEG engager^TfR^-decorated Doxisome. (A) Schematic diagram of anti-GBM therapies of pH-PEG engager^TfR^-decorated Doxisome. (B) DNS engager^TfR^, WT-PEG engager^TfR^, or pH-PEG engager^TfR^ was premixed with Doxisome. Groups of six or eight BALB/c nude mice bearing intracranial U-87 MG-Luc2 GBM were intravenously injected with PBS alone, DNS engager^TfR^-, WT-PEG engager^TfR^-, or pH-PEG engager^TfR^-decorated Doxisome (3 mg kg^–1^) once a week for 3 weeks. The bioluminescence corresponding to GBM growth was monitored by an IVIS Spectrum imaging system weekly. The representative bioluminescence images were shown. (C) Anti-GBM treatments show mean bioluminescence of tumors at 6 days and 27 days post GBM implantation. Data are shown as mean ± standard deviation. Statistical analysis of the differences in tumor growth between treatment and control groups was performed by one-way ANOVA; *, *p* ≤ 0.0457, ****, *p* ≤ 0.0001. (D) Kaplan–Meier survival analysis of GBM mice. Median survival time of different treated groups is indicated in which 47.5, 56, 52, and 84 days for PBS, DNS engager^TfR^, WT-PEG engager^TfR^, or pH-PEG engager^TfR^ groups, respectively. Significant differences in median survival time between PBS alone, DNS engager^TfR^-, WT-PEG engager^TfR^-, and pH-PEG engager^TfR^-decorated Doxisome-treated groups are indicated: *, *p* = 0.0427, **, *p* = 0.0021, ***, *p* ≤ 0.0009 (Kaplan–Meier analysis, log-rank test). n.s., not significant.

Functionalized liposomal doxorubicin has been widely used for the treatment of glioblastoma in preclinical studies.^49^ Therefore, we further investigated the efficacy of the pH-PEG engager^TfR^-decorated Doxisome in an orthotopic human GBM mouse model. DNS engager^TfR^, WT-PEG engager^TfR^, or pH-PEG engager^TfR^ was premixed with Doxisome to generate 60-engager^TfR^-Doxisomes (60 engagers per Doxisome). BALB/c nude mice bearing intracranial U-87 MG-Luc2 GBM implants were intravenously injected with PBS alone, DNS engager^TfR^-, WT-PEG engager^TfR^-, or pH-PEG engager^TfR^-decorated Doxisome (3 mg kg^–1^) on days 6, 13, and 20 post orthotopic GBM implantation (Figure 6A). The whole-body bioluminescence imaging was detected by an IVIS Spectrum imaging system weekly (Figures 6B and S12). Figure 6C showed that the bioluminescence of GBM in mice revealed no significant differences among the groups before treatments (day 6), while pH-PEG engager^TfR^-decorated Doxisome exhibited significant GBM tumor suppression as compared to DNS engager^TfR^ or WT-PEG engager^TfR^ control groups at 27 days post orthotopic GBM implantation. Additionally, Kaplan–Meier survival analysis showed that pH-PEG engager^TfR^-decorated Doxisome significantly prolonged the survival of GBM-bearing mice with a median survival time of 84 days (*p* < 0.05). By contrast, PBS or DNS engager^TfR^ or WT-PEG engager^TfR^ control groups displayed similar short median survival times of 47.5, 56, and 52 days, respectively, without significant differences (*p* > 0.05) (Figure 6D). These results suggested that pH-PEG engager^TfR^ markedly enhanced the delivery of PEGylated Doxisomes across the BBB in GBM mice, leading to an improved anti-GBM efficacy. Notably, even though WT-PEG engager^TfR^-decorated PEG-NPs can specifically target TfR-expressing cells (Figure 3), it is not beneficial for extending the survival rate of GBM-bearing mice, indicating that pH-PEG engager^TfR^ is crucial for efficient PEGylated Doxisome dissociation in acidic endosomes of BMECs to facilitate PEGylated nanomedicine delivery across the BBB.

## Discussion

We have shown that one-step mixing of pH-PEG engager^TfR^ with PEGylated nanomedicine forms stable TfR-targeting complexes under physiological pH and subsequently dissociates under acidic pH conditions. pH-PEG engager^TfR^-decorated PEG-LPs efficiently traversed the BBB in vitro, presumably due to the activation of TfR-mediated transcytosis in BMECs at physiological pH followed by efficient dissociation of PEG-LPs at acidic endosomes, which might be beneficial for greater PEG-LPs delivery across the BBB. pH-PEG engager^TfR^ significantly enhanced the accumulation of PEG-LPs in mouse brains. Most importantly, pH-PEG engager^TfR^-mediated Doxisome treatment markedly suppressed orthotopic GBM tumor growth and greatly extended the survival of GBM-bearing mice.

Previous studies have reported that modulating the affinity or pH-responsive binding of antibodies against BBB-related TfR can significantly facilitate the efficient payload release via receptor-mediated transcytosis to enhance drug delivery across the BBB.^19−21^ However, functionalizing the surface of nanomedicine with BBB-permeable ligands, such as glucose, transferrin, or low-affinity TfR antibodies, requires complicated optimization of ligand density since an excess of ligand density might be compensated by high-avidity effects leading to inefficient payload release and then reduce drug delivery across the BBB.^50−52^ Although pH-sensitive anti-TfR antibodies might overcome the problem of avidity effects on the nanomedicine,^21^ pH-responsive ligands for newly explored BBB-related receptors typically need to be developed on a case-by-case basis. By contrast, pH-PEG engagers are compatible with any PEGylated therapeutics for conditional cargo dissociation in acidic endosomes after transcytosis and remarkably, are easily switchable between different ligands targeting novel BBB-related receptors.^53^

Although pH-PEG engager^TfR^ exhibited enhanced brain delivery of PEGylated nanomedicine to prolong the survival rate of orthotopic GBM mice significantly, none of these treated mice were cured, indicating the need for specific targeting of nanomedicine to GBM after crossing the BBB to improve the therapeutic index. Several studies have found that 60–84% of GBM cancers overexpress epidermal growth factor receptors.^54,55^ Therefore, a cocktail of WT-PEG engager^EGFR^ and pH-PEG engager^TfR^ could be simply utilized for brain delivery of PEGylated drugs followed by specific targeting of EGFR-overexpressing GBM. Doxorubicin has been reported as an immunogenic cell death inducer that enhance cancer immunotherapy for many cancers, including GBM.^56,57^ In fact, the uptake of PEGylated liposomal doxorubicin in brain stimulated greater presentation of tumor antigens to T cells, higher INF-γ production by microglia and led to improved therapeutic response to programmed cell death-1 (PD-1) blockade therapy in GBM.^58^ Moreover, numerous reports demonstrated that GBM cells express programmed cell death ligand-1 (PD-L1), thereby PD-1/PD-L1 immune checkpoint blockade therapies revealed enhanced anti-GBM efficacy.^59,60^ Thus, combined WT-PEG engager^PD-L1^ and pH-PEG engager^TfR^ with PEGylated liposomal doxorubicin presumably increases drug accumulation in GBM to induce tumor immunogenicity as well as inhibit PD-1/PD-L1 immune evasion of GBM.

The pH-PEG engager platform might also improve the treatment of LSDs. LSDs are a group of genetic disorders, each caused by the deficiency of specific lysosomal enzymes responsible for glycan catabolism, leading to the accumulation of undigested substrates in lysosomes and subsequent cell death and tissue damage.^61^ Enzyme replacement therapies resupply the recombinant lysosomal enzymes that are lacking in LSD patients. Even though LSDs are not CNS disorders, they involve progressive CNS dysfunction since BBB hindered most enzyme replacement therapies.^61,62^ Therefore, the pH-PEG engagers coupled with PEGylated lysosomal enzymes such as Pegunigalsidase alfa^63^ or PEG-LNP-lysosomal enzyme mRNA^64,65^ may enhance the uptake or expression of lysosomal enzymes in the brain to suppress the progression of LSD-related neurodegeneration.

Effective active targeting of PEGylated nanomedicine by using bispecific PEG engagers has been developed for targeted cancer therapies.^28,29^ Two kinds of PEG engager-mediated delivery strategies were employed. First, one-step predecoration strategy by tethering PEG engagers to the PEGylated nanomedicine can confer cancer-selectivity of PEGylated nanomedicine to cancers without the need of complicated chemical conjugation procedures.^29^ Second, two-step pretargeted strategy involves the first administration of PEG engagers to bind target cells followed by PEGylated nanomedicine injection after the clearance of excess circulating PEG engagers. The two separate administrations of PEG engagers and PEGylated nanomedicine ensure that their original properties are not influenced as compared to predecoration strategy. One pivotal aspect for pretargeted strategy is that PEG engagers should remain dormant on the target cell membrane until interacting with PEGylated nanomedicine and trigger endocytosis into target cells. However, the TfR-targeting domain of pH-PEG engager^TfR^ used in this study can directly stimulate TfR-mediated transcytosis,^41^ indicating that pretargeted strategy is not appropriate in this study. Therefore, predecoration strategy was chosen to test whether pH-PEG engager^TfR^-decorated PEG-LPs facilitate the delivery of PEG-LPs across the BBB.

## Conclusions

In summary, we demonstrated that pH-PEG engager^TfR^ can greatly facilitate the BBB penetration of the PEGylated nanomedicine and suppress the growth of orthotopic GBM in mice. Thus, we believe this simple and switchable BBB-shuttle strategy may serve as a versatile approach for enhanced brain delivery of PEGylated medicines, including drug-loaded nanoparticles, therapeutic proteins, or LNP-mRNAs to improve the treatment of CNS diseases.

## Methods

### Cell Lines and Animals

All cells were cultured in Dulbecco’s modified Eagle’s medium (Sigma-Aldrich, St Louis, MO) containing 10% heat-inactivated fetal bovine serum (HyClone, Logan, Utah), 100 μg mL^–1^ streptomycin and 100 U mL^–1^ penicillin at 37 °C in a humidified atmosphere of 5% CO_2_ in air unless otherwise mentioned. Human U-87 MG-Luc2 glioblastoma cell line and mouse brain endothelial cell line bEnd.3 were obtained from the American Type Culture Collection (Manassas, VA). 293-hTfR and 293-hTfR-GFP cells were generated by lentiviral transduction of the human TfR gene and human TfR-green fluorescent protein (GFP) fusion gene into 293FT cells, respectively (Thermo Fisher Scientific, San Jose, CA). The human BMEC line, hCMEC/D3, was purchased from Merck and cultured in EndoGRO-MV Complete Media Kit containing 1 ng mL^–1^ of fibroblast growth factor 2 (Sigma-Aldrich, St Louis, MO). ExpiCHO-S cells were purchased from Thermo Fisher Scientific and were cultured in ExpiCHO Expression Medium (Thermo Fisher Scientific, San Jose, CA) at 37 °C in a humidified atmosphere of 8% CO_2_ in air. Healthy 6–8 week old female BALB/c nude mice (BALB/cAnN.Cg-*Foxn1*^*nu*^/CrlNarl) were purchased from the National Laboratory Animal Center, Taipei, Taiwan and were maintained under specific pathogen-free conditions at the National Yang Ming Chiao Tung University Laboratory Animal Center. All animal studies were performed in accordance with guidelines and ethically approved by the institutional animal care and use committee of the National Yang Ming Chiao Tung University.

### Generation of pH-Responsive PEG Engager Variants by Site-Directed Mutagenesis

The wild-type V_L_-Cκ and V_H_-CH_1_-hexahistidine-tag DNA fragments of humanized anti-PEG 6.3 (h6.3) Fab were inserted into a pKM vector containing the pelB and the stII signal peptides for periplasm expression in *E. coli*. The h6.3 Fab (PEG engager) variants were mutated using site-directed mutagenesis^66^ for histidine substitution of residues corresponding to h6.3 dimerization. The C43 (DE3) *E. coli* that harbor PEG engager variants were cultured in 2 × YT Broth at 37 °C until reaching an OD600 value of 0.5 and then were induced with 1 mmol L^–1^ of isopropyl β-D-1-thiogalactopyranoside at 30 °C for 20 h. The cells were harvested by centrifugation at 3000*g* for 15 min at 4 °C, and the pellet was lysed using the B-PER reagent (Thermo Fisher Scientific, San Jose, CA). The crude protein solution of PEG engager variants was purified by using His SpinTrap TALON (Cytiva, Marlborough, MA) and buffer exchanged in PBS for the pH-dependent PEG-binding enzyme-linked immunosorbent assay (ELISA). The mutated residues of PEG engager variants possessing pH-responsive PEG-binding activity were combined to generate the pH-PEG engager.

### Production of Recombinant Bispecific DNS, WT-PEG, and pH-PEG Engager

The V_L_-Cκ and V_H_-CH_1_ DNA fragments of anti-DNS or wild-type PEG engager or pH-PEG engager were joined by an internal ribosome entry site element and then inserted into the pLPCX plasmid to generate DNS engager, WT-PEG engager, and pH-PEG engager DNA vectors. The synthetic TfRB1G3 DNA fragment (GenScript, Piscataway NJ) was further cloned into DNS engager, WT-PEG engager, and pH-PEG engager plasmids to generate DNS engager^TfR^, WT-PEG engager^TfR^, and pH-PEG engager^TfR^. ExpiCHO-S cells were transfected with DNS engager^TfR^, WT-PEG engager^TfR^, and pH-PEG engager^TfR^ DNA plasmids using an ExpiFectamine CHO Transfection Kit (Thermo Fisher Scientific, San Jose, CA). The medium was collected 10 days post-transfection by centrifugation at 1000*g* for 5 min and then filtered through a 0.45 μm filter. Polyhistidine-tagged DNS engager^TfR^, WT-PEG engager^TfR^, and pH-PEG engager^TfR^ proteins were purified on a HiTrap TALON crude column (Cytiva, Marlborough, MA) and the concentration of these protein samples was determined using a bicinchoninic acid protein assay (ThermoFisher Scientific, San Jose, CA). Five micrograms of purified DNS engager^TfR^, WT-PEG engager^TfR^ or pH-PEG engager^TfR^ were electrophoresed in a 12.5% SDS-PAGE gel under reducing or nonreducing conditions and then stained by Coomassie Blue.

### Preparation of PEG-LPs

To prepare fluorescent PEG-LPs (PEG-lipoDiR, PEG-lipoDiD, and PEG-lipoDiO), Distearoylphosphatidylcholine (DSPC), 1,2-distearoyl-*sn*-glycero-3-phosphoethanolamine-*N*-[methoxy(polyethylene glycol)-2000 (DSPE-mPEG_2000_), cholesterol (Avanti Polar Lipids, Inc.), and fluorescent dyes: 1,1-dioctadecyl-3,3,3,3-tetramethylindotricarbocyanine iodide (DiR) or 1,1-dioctadecyl-3,3,3,3-tetramethylindodicarbocyanine (DiD) or 3,3-dioctadecyloxacarbocyanine perchlorate (DiO) (AAT Bioquest, Pleasanton, CA) were dissolved in chloroform at a 65:5:30:0.05 molar ratio, respectively. To prepare amino-PEG-LPs for anti-PEG ELISAs, DiR, DiD and DiO were excluded, and DSPE-mPEG_2000_ was replaced by DSPE-PEG_2000_-Amine (Avanti Polar Lipids, Inc.). The mixed lipids in chloroform were dried by rotary evaporation at 65 °C and rehydrated in Tris-buffered saline (TBS, 50 mmol L^–1^ Tris–HCl, 150 mmol L^–1^ NaCl, pH 7.4) or H_2_O for fluorescent PEG-LPs and amino-PEG-LPs, respectively. The lipid suspension was repeatedly incubated in liquid nitrogen and a heated water bath at 80 °C for 10 cycles. The freeze–thawed liposomal suspension was sequentially extruded through 400, 200, and 100 nm polycarbonate membranes at 75 °C 21 times per membrane using a mini-extruder (Avanti Polar Lipids, Inc.). Doxisome was kindly provided by Taiwan Liposome Company. The final lipid concentration of PEG-LPs and Doxisome was measured by Bartlett’s assay^67^ and adjusted to 14 and 16.7 mmol L^–1^, respectively. The doxorubicin concentration of the Doxisome is 2 mg mL^–1^.

### pH-Dependent ELISA

Five μmol L^–1^ of amino-PEG-LPs prepared in 100 mmol L^–1^ NaHCO_3_/Na_2_CO_3_ coating buffer (pH 8.0) were added to Maxisorp 96-well microplates (50 μL per well) (Thermo Fisher Scientific, San Jose, CA) for 3 h at 37 °C and then blocked with 250 μL of 5% (wt/vol) skim milk in PBS at 4 °C overnight. Purified PEG engager variants or graded concentrations of purified DNS engager^TfR^, WT-PEG engager^TfR^, or pH-PEG engager^TfR^ in 50 μL of 5% (w/v) skim milk in PBS (pH 7.4) were added to the plates at room temperature (RT) for 1 h. Unbound proteins were washed thrice with PBS (pH 7.4) or citrate buffer (pH 5.8). The plates were incubated with 1 μg mL^–1^ of HPR-conjugated goat anti-human F(ab’)_2_ antibodies (Jackson Immuno Research Laboratories, West Grove, PA) in 50 μL of 5% (wt/vol) skim milk at RT for 30 min. After washing with PBS three times, bound peroxidase activity was measured by adding 150 μL per well of ABTS substrate solution (0.4 mg mL^–1^ 2,2′-azino-di (3-ethylbenzthiazoline-6-sulfonic acid) (Sigma-Aldrich, St. Louis, MO), 0.003% H_2_O_2_, 100 mmol L^–1^ phosphate citrate, pH 4.0) for 30 min at RT. The absorbance (405 nm) was measured in a SpectraMax ABS Plus microplate reader (Molecular Device, Menlo Park, CA). GraphPad Prism 6 was used to analyze the ELISA data.

### Preparation of PEG Engager^TfR^-Decorated PEG-LPs

All of the PEG-LPs used in this study possess the same lipid composition (DSPC/DSPE-mPEG_2000_/cholesterol = 65:5:30). The bispecific antibodies, including DNS engager^TfR^, WT-PEG engager^TfR^, or pH-PEG engager^TfR^ were mixed with PEG-LPs at different antibody-to-DSPE-mPEG_2000_ molar ratios of 1:330, 3:330, 6:330, and 18:330 at 4 °C for 1 h. Based on the formula established by Kirpotin et al.,^68^ a 95 nm PEG-LP contains ∼71,865 phospholipid molecules and ∼3593 DSPE-mPEG_2000_. Therefore, the corresponding number of PEG engager^TfR^ per PEG-LPs was estimated to be 10, 30, 60 and 180, respectively.

### Flow Cytometer Analysis

DNS engager^TfR^, WT-PEG engager^TfR^, or pH-PEG engager^TfR^ were premixed with PEG-lipoDiD at different antibody-to-DSPE-mPEG_2000_ molar ratios of 1:330, 3:330, 6:330, and 18:330 in PBS (pH7.4) at RT for 30 min (1.65 μg of engager protein and 0.05 mmol L^–1^ of PEG-lipoDiD). 293-mTfR cells were stained with DNS engager^TfR^- or WT-PEG engager^TfR^- or pH-PEG engager^TfR^-decorated PEG-lipoDiD (50 μmol L^–1^ of total lipid concentration) in staining buffer (PBS containing 0.1% bovine serum albumin, pH 7.4) for 1 h at 4 °C. The cells were washed with cold PBS (pH 7.4) or MES-buffered saline (25 mmol L^–1^ MES, 150 mmol L^–1^ NaCl, pH 5.8) three times and the surface fluorescence of 10^4^ viable cells was measured by Guava easyCyte Flow Cytometer (Cytek Biosciences) and analyzed with Flowjo (Tree Star Inc.).

### Fluorescence Microscopy Analysis

293-mTfR cells (3 × 10^5^ cells per well) were seeded overnight on the 6-well culture plates. DNS engager^TfR^- or WT-PEG engager^TfR^- or pH-PEG engager^TfR^-decorated PEG-lipoDiO (antibody-to-DSPE-mPEG_2000_ molar ratio = 6:330) were diluted in culture medium (DMEM, 10% FBS) to a final lipid concentration of 50 μmol L^–1^ and supplied with 1 μg mL^–1^ of Hoechst 33342 (ThermoFisher Scientific) for staining with 293-mTfR cells at 37 °C for 30 min. The cells were washed with free DMEM (pH 7.4) or DMEM supplied with 5 mmol L^–1^ MES (adjusted with HCl to pH 5.8) three times and were visualized on a ZOE Fluorescent Cell Imaging System (Bio-Rad Laboratories, Inc.) at excitation and emission wavelengths of 355/40 and 433/36 nm for Hoechst 33342 and 480/17 and 517/23 nm for PEG-lipoDiO.

### In Vitro BBB Transwell Assay

bEnd.3 or hCMEC/D3 cells (10,000 cells per well) were seeded on polycarbonate 24-well transwell membranes with mean pore size of 0.4 μm and surface area of 0.33 cm^2^ (Corning) for monolayer cell culture until the transendothelial electrical resistance detected by an epithelial voltmeter (Millicell-RES, Millipore, USA) is greater than 40 Ω. For the pulse-chase assay, DNS engager^TfR^-, WT-PEG engager^TfR^-, or pH-PEG engager^TfR^-decorated PEG-LPs (antibody-to-DSPE-mPEG_2000_ molar ratio = 3:330, 6:330, or 18:330) were diluted in culture medium to a final lipid concentration of 0.7 mmol L^–1^ and added to the upper chamber of transwell plates (100 μL) for pulse 1 h. For the chase phase, both upper and lower compartments were washed twice with culture medium and then incubated for 2 h. Finally, the medium was collected from the lower compartments and analyzed using quantitative anti-PEG sandwich ELISAs.^43−45^ Maxisorp 96-well microplates were coated with 0.25 μg of AGP4 anti-PEG IgM per well in 50 μL of 100 mmol L^–1^ NaHCO_3_/Na_2_CO_3_ coating buffer (pH 8.0) for 3 h at 37 °C and then blocked with 250 μL of 5% (w/v) skim milk in PBS at 4 °C overnight. Graded concentrations of PEG-LPs or collected samples (50 μL per well) were added to the plates at RT for 2 h. After washing by PBS six times, the plates were sequentially stained with 5 μg mL^–1^ of biotinylated 3.3 anti-PEG IgG and HRP-conjugated streptavidin (1 μg mL^–1^, Jackson Immuno Research Laboratories, West Grove, PA) in 50 μL of 2% (w/v) skim milk at RT for 1 h. The plates were washed by PBS eight times, and bound peroxidase activity was measured by adding 150 μL per well of ABTS substrate solution (0.4 mg mL^–1^ 2,2′-azino-di (3-ethylbenzthiazoline-6-sulfonic acid) (Sigma-Aldrich, St. Louis, MO), 0.003% H_2_O_2_, 100 mmol L^–1^ phosphate citrate, pH 4.0) for 30 min at RT. The absorbance (405 nm) was measured in a SpectraMax ABS Plus microplate reader (Molecular Device, Menlo Park, CA).

### IVIS Imaging and Immunohistochemistry Staining

BALB/c nude mice were intravenously injected with 100 μL of 5 mmol L^–1^ of DNS engager^TfR^- or WT-PEG engager^TfR^- or pH-PEG engager^TfR^-decorated PEG-lipDiR (antibody-to-DSPE-mPEG_2000_ molar ratio = 6:330, 500 nmol of PEG-lipDiR per mouse), respectively. Isoflurane anesthetized mice were imaged with an IVIS Spectrum imaging system (excitation, 745 nm; emission, 840 nm; PerkineElmer) 0.5, 6, and 24 h after injection. These mice were also sacrificed and perfused with 50 mL of PBS at 24 h after injection. The organs were collected for IVIS imaging. For immunohistochemistry staining, the isolated brain organs were fixed in 10% PBS-buffered formalin and embedded in paraffin. The brain sections (4 μm) were dewaxed with xylene, rehydrated to water, treated with sodium citrate buffer (10 mmol L^–1^ sodium citrate, 0.05% Tween 20, pH 6.0) for antigen retrieval, and followed by bovine serum albumin blocking buffer (5% w/v in PBS) incubation. The brain sections were stained with 5 μg mL^–1^ of biotinylated AGP4 anti-PEG IgM in staining buffer at 4 °C overnight followed by HRP-conjugated streptavidin (1 μg mL^–1^) at RT for 1 h. Unbound antibodies were removed by washing with PBS and then bound peroxidase activity was measured by adding DAB substrate for 15 min at RT. Stained sections were counterstained with hematoxylin, mounted with VectaMount Express Mounting Medium (Vector Laboratories, Burlingame, CA), and observed under a light microscope.

### In Vivo Anti-GBM Therapy

To perform orthotopic implantation of GBM, isoflurane-anesthetized BALB/c nude mice were positioned into the ear bars of a Digital Stereotaxic Instruments (RWD, Sugar Land, TX) and a 0.8 cm incision was made using a sterile scalpel to expose the bregma. A burr hole was generated at the position 2 mm posterior and 1.5 mm lateral to the bregma in the right cerebral hemisphere, and 5 × 10^4^ U-87 MG-Luc2 GBM cells in 6 μL of sterile PBS were injected 2.5 mm deep from the dura at a rate of 0.5 μL/min using a Legato 130 Syringe Pump (KD Scientific). After intracranial injection of GBM cells, the burr holes were closed by applying bone wax and followed by veterinary tissue glue (3 M Vetbond) application to seal the wound. The orthotopic GBM mice were intravenously injected with PBS alone or DNS engager^TfR^- or WT-PEG engager^TfR^- or pH-PEG engager^TfR^-decorated Doxisome (3 mg kg^–1^, antibody-to-DSPE-mPEG_2000_ molar ratio = 6:330) on days 6, 13, and 20 post orthotopic GBM implantation. The GBM growth was determined weekly by intraperitoneal injection of D-luciferin (150 mg kg^–1^) to the mice 20 min before bioluminescence imaging on an IVIS system.

### Statistical Analysis

Results are presented as the mean ± standard deviation. Statistical analyses and figures were generated using GraphPad Prism 6. Significance among groups is determined using one-way ANOVA, two-way ANOVA, and log-rank test, according to test requirements. A probability value < 0.05 was considered statistically significant (*p < 0.05, **p < 0.01, ***p < 0.001, and ****p < 0.0001).

## Supporting Information Available

Information and methods; cocrystal structure of anti-PEG h6.3 Fab and PEG, and their X-ray data collection and refinement statistics; cross-reactivity of TfRB1G3 against to mTfR and hTfR; coupling efficacy of PEG engager-decorated PEG-LPs; physicochemical characterization of PEG engager-decorated PEG-LPs; stability of PEG engager-decorated PEG-LPs; analysis of colocalization of PEG engager^TfR^-decorated PEG-LPs with TfR in cells; pH-PEG engager^TfR^ enhances brain uptake of PEG-NPs in GBM-bearing mice; pharmacokinetics of PEG engagers and PEG-LPs in mice; and anti-GBM efficacy of pH-PEG engager^TfR^-decorated Doxisome (PDF)
