# Supplementary material for: pH-Responsive Polyethylene Glycol Engagers for Enhanced Brain Delivery of PEGylated Nanomedicine to Treat Glioblastoma
Source: ACS Nano. 2025 Jan 3;19(1):307–21. doi: 10.1021/acsnano.4c05906 (PMC11752499; doi:10.1021/acsnano.4c05906)
Supplement: Supplementary file 1 — nn4c05906_si_001.pdf [file nn4c05906_si_001.pdf]

Supporting Information

pH-Responsive Polyethylene Glycol Engagers for Enhanced Brain Delivery of PEGylated Nanomedicine to Treat Glioblastoma

Jun-Lun Meng[1, #], Zi-Xuan Dong[1, #], Yan-Ru Chen[1, #], Meng-Hsuan Lin[1], Yu-Ching Liu[1], Steve R. Roffler[2, 3], Wen-Wei Lin[4], Chin-Yuan Chang[1], Shey-Cherng Tzou[1, 5], Tian-Lu Cheng[5], Hsiao-Chen Huang[1], Zhi-Qin Li[1], Yen-Cheng Lin[1], and Yu-Cheng Su[1, 5]*

[1]Department of Biological Science and Technology, Center for Intelligent Drug Systems and Smart Bio-devices (IDS[2]B), National Yang Ming Chiao Tung University, Hsinchu, 300, Taiwan.

[2]Institute of Biomedical Sciences, Academia Sinica, Taipei, 115, Taiwan.

[3]Graduate Institute of Medicine, College of Medicine, Kaohsiung Medical University, Kaohsiung, 807, Taiwan

[4]School of Post-Baccalaureate Medicine, College of Medicine, Kaohsiung Medical University, Kaohsiung, 807, Taiwan.

[5]Department of Biomedical Science and Environmental Biology, Drug Development and Value Creation Research Center, Kaohsiung Medical University, Kaohsiung, 807, Taiwan.

[#]Jun-Lun Meng, Zi-Xuan Dong, and Yan-Ru Chen contributed equally to this work

*Correspondence to:
Dr. Yu-Cheng Su, Department of Biological Science and Technology, National Yang Ming Chiao Tung University, Room 720, BioICT Building, 75 Bo-Ai Street, Hsinchu, Taiwan.
Tel: (886)-3-5712-121 ext. 59733
Fax: (886)-3-5729-288
Email: ycsu-johnny@nycu.edu.tw

Materials and Methods

Crystallization and data collection

Humanized anti-PEG h6.3 Fab in complex with PEG (h6.3 Fab/PEG) was crystallized using the hanging-drop vapor diffusion method. The h6.3 Fab was buffer exchanged in 20 mmol L^{-1} Tris containing 100 mmol L^{-1} NaCl, pH 7.5, and concentrated to 18 mg mL^{-1}. The complex crystals of the anti-PEG h6.3 Fab/PEG were grown by mixing 1 μL protein solution (18 mg mL^{-1}) with 1 μL reservoir solution at 20 °C. The anti-PEG h6.3 Fab/PEG crystals were crystallized under a screen condition: 18% (w/v) PEG-6000, 1% (w/v) PEG-2000 methyl ether, 0.15 mol L^{-1} lithium sulfate monohydrate, and 0.1 mol L^{-1} citric acid (pH 3.5). Snap-freezing the crystals with 20% glycerol (v/v) as a cryoprotectant for X-ray data collection at cryogenic temperatures (80 K). The diffraction data of the anti-PEG h6.3 Fab/PEG crystals were collected at the National Synchrotron Radiation Research Center (NSRRC, Taiwan) on beamline BL13B1 using a wavelength of 1 Å with the ADSC QUANTUM 315r CCD detector (Area Detector Systems Corporation, Poway, CA). Data were indexed and scaled using HKL2000.

Structure determination and refinement

The crystal structure of anti-PEG h6.3 Fab/PEG was determined by the molecular replacement method of MOLREP using the structure of the mouse anti-PEG antibody 6.3 fragment in complex with PEG (Protein Data Bank entry 6VL9) as a search model.[1] Extensive manual model building and refinement were performed using COOT. The models were further refined with REFMAC. The final model shows 1.66% outliers and 93.35% of residues in the most favored regions and 4.99% of residues in the additionally allowed regions of the Ramachandran diagram. The atomic coordinates and structure factors of anti-PEG h6.3 Fab/PEG were deposited in the Protein Data Bank as entry 8Z95. PyMol was used to generate figures of structures.

Flow cytometer analysis

293-mTfR or 293-hTfR cells were stained with APC-conjugated anti-mTfR antibodies or APC-conjugated anti-hTfR antibodies (BioLegend, San Diego, CA) to determine the expression levels of mTfR and hTfR on the cells. DNS engager^{TfR}, WT-PEG engager^{TfR}, WT-PEG engager^{CD19}, or pH-PEG engager^{TfR} were pre-mixed with

PEG-lipoDiD at different antibody-to-DSPE-$mPEG_{2000}$ molar ratios of 3:330, and 6:330 in PBS (pH7.4) at RT for 30 min (1.65 μg of engager protein and 0.05 mmol L^{-1} of PEG-lipoDiD) freshly or incubation for 3 days. 293-mTfR or 293-hTfR cells were stained with freshly prepared or 3-days incubated DNS $engager^{TfR}$ or WT-PEG $engager^{TfR}$ or pH-PEG $engager^{TfR}$ decorated PEG-lipoDiD (50 μmol L^{-1} of total lipid concentration) in staining buffer (PBS containing 0.1% bovine serum albumin, pH 7.4) for 1 h at 4°C. These immunostained cells were washed with cold PBS (pH 7.4) three times and the surface fluorescence of 10^4 viable cells was measured by Guava® easyCyte Flow Cytometer (Cytek Biosciences) and analyzed with Flowjo (Tree Star Inc.).

Sandwich ELISAs

Maxisorp 96-well microplates were coated with 0.15 μg of anti-6 × His tag antibodies (iReal Biotechnology, Taiwan) (for the quantification of DNS and PEG engagers) or 0.25 μg of AGP4 anti-PEG IgM (for PEG-LPs measurement) per well in 50 μL of 100 mmol L^{-1} $NaHCO_3/Na_2CO_3$ coating buffer (pH 8.0) for 3 h at 37 °C and then blocked with 250 μL of 5% (wt/vol) skim milk in PBS at 4 °C overnight. Graded concentrations of purified PEG engagers or collected samples or PEG-LPs (50 μL per well) were added to the plates at RT for 2 h. After washing by PBS six times, the plates were sequentially stained with 1 μg mL^{-1} of biotinylated anti-human Ck antibodies (Vector Labs, Burlingame, CA) (for the quantification of DNS and PEG engagers) or 5 μg mL^{-1} of biotinylated 3.3 anti-PEG IgG (for PEG-LPs measurement) and followed by HRP-conjugated streptavidin (1 μg mL^{-1}, Jackson Immuno Research Laboratories, West Grove, PA) in 50 μL of 2% (wt/vol) skim milk at RT for 1 h. The plates were washed by PBS eight times and bound peroxidase activity was measured by adding 150 μL per well of ABTS substrate solution (0.4 mg mL^{-1} 2,2'-azino-di (3-ethylbenzthiazoline-6-sulfonic acid) (Sigma–Aldrich, St. Louis, MO), 0.003% H_2O_2, 100 mmol L^{-1} phosphate citrate, pH 4.0) for 30 min at RT. The absorbance (405 nm) was measured in a SpectraMax ABS Plus microplate reader (Molecular Device, Menlo Park, CA).

Conjugation rates of PEG-$engager^{TfR}$-PEG-LPs

WT-PEG $engager^{TfR}$ or pH-PEG $engager^{TfR}$ were pre-mixed with PEG-lipoDiD at different antibody-to-DSPE-$mPEG_{2000}$ molar ratios of 1:330, 3:330, 6:330 and

18:330 in PBS (pH7.4) at RT for 30 min (1.65 μg of engager protein and 0.05 mmol L^{-1} of PEG-lipoDiD). These PEG engager^{TfR} decorated PEG-lipoDiDs were pallet down by ultracentrifugation 100,000g for 2 h using Optima MAX-XP ultracentrifuge (Beckman Coulter, South Kraemer Boulevard Brea, CA) and the supernatants were collected. The PEG engager levels in supernatants were determined by quantitative anti-His/ anti-human Fab sandwich ELISA, as described above. The coupling rates of PEG engager decorated PEG-LPs were calculated according the formula: (total amount of PEG engagers – free PEG engagers) / total amount of PEG engagers × 100.

Characterization of PEG-engager^{TfR}-PEG-LPs

The mean particle size and zeta potential of the PEG-LPs before and after engager^{TfR} (DNS engager^{TfR}, WT-PEG engager^{TfR}, or pH-PEG engager^{TfR}) complexation at room temperature were analyzed by using dynamic light scattering (Zetasizer nano ZS, Malvern, UK).

Confocal microscopy

Glass coverslips (30 mm) were coated with poly-L-lysine (10 μg mL^{-1}) in PBS for 30 min at RT and then assembled in the chamber of POCmini cultivation system (perfusion open and closed). 293-hTfR-GFP cells (5×10^4 cells per coverslip) were seeded on the coverslips overnight. WT-PEG engager^{TfR} or pH-PEG engager^{TfR} decorated PEG-lipoDiD (antibody-to-DSPE-$mPEG_{2000}$ molar ratio = 6:330) were prepared in culture medium (DMEM, 10% FBS) to a final lipid concentration of 50 μmol L^{-1} for staining with 293-hTfR-GFP cells at 37°C for 1 h. The cells were washed with culture medium twice and were imaged on an Axiovert 200M Confocal Microscope (Carl Ziess Inc.) at excitation and emission wavelengths of 488 and 525 nm for GFP and 650 and 675 nm for DiD at 37 °C, 5% CO_2.

Pharmacokinetics analysis

BALB/c nude mice were intravenously injected with 45 μg of DNS engager^{TfR}, WT-PEG engager^{TfR}, or pH-PEG engager^{TfR} with or without pre-docking to Doxisome (antibody-to-DSPE-$mPEG_{2000}$ molar ratio: 6:330). Blood samples were periodically collected from the tail vein of the mice and plasma was isolated d by centrifugation (12,000g, 5 min). The PEG engager and Doxisome levels in plasma were determined by quantitative anti-His/ anti-human Fab and anti-PEG sandwich ELISA, respectively

as described above. The half-lives of the DNS engagers, or PEG engagers, or Doxisomes were estimated by fitting the data to a one-phase exponential decay model with GraphPad Prism 6 software.

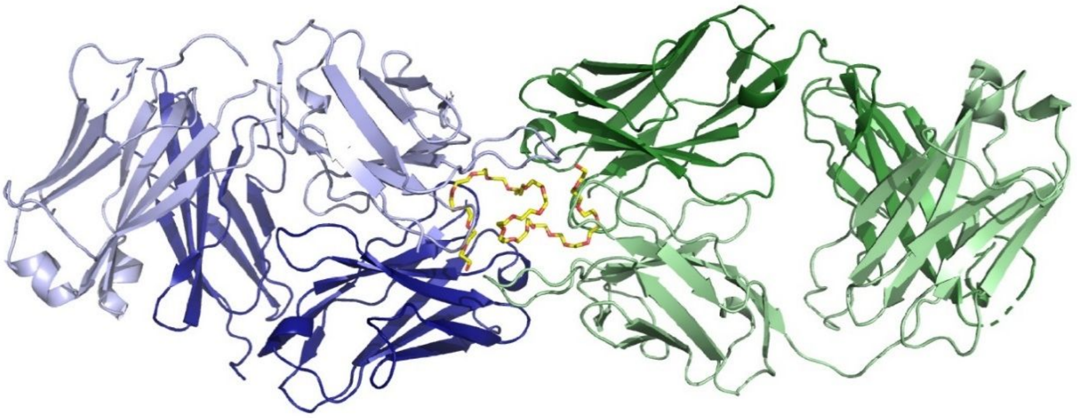

Figure S1. The crystal structure of humanized anti-PEG h6.3 Fab in complex with PEG. The two Fab subunits are colored green and blue. The heavy chain and light chain of Fab are colored in deep and light color, respectively. PEG molecule is shown as yellow sticks.

Table S1. Data collection and refinement statistics.

	**h6.3 Fab**
**Data collection**	
Space group	C2
Cell dimensions	
a, *b*, *c* (Å)	245.37, 43.57, 110.15
α, β, γ (°)	90.00, 111.70, 90.00
Resolution (Å)	30.00–2.27 (2.35–2.27)
R_{sym} or R_{merge}	4.3 (33.3)
I / σ	29.1 (3.9)
Completeness (%)	99.1 (99.2)
Redundancy	3.6 (3.7)
**Refinement**	
Resolution (Å)	30.00–2.27
No. reflections	640383
R_{work} / R_{free}	0.209/0.262
No. atoms	
Protein	6540
PEG	70
Water	180
B-factors	
Protein	39.6
PEG	29.9
Water	31.2
R.m.s. deviations	
Bond lengths (Å)	0.0084
Bond angles (°)	1.49

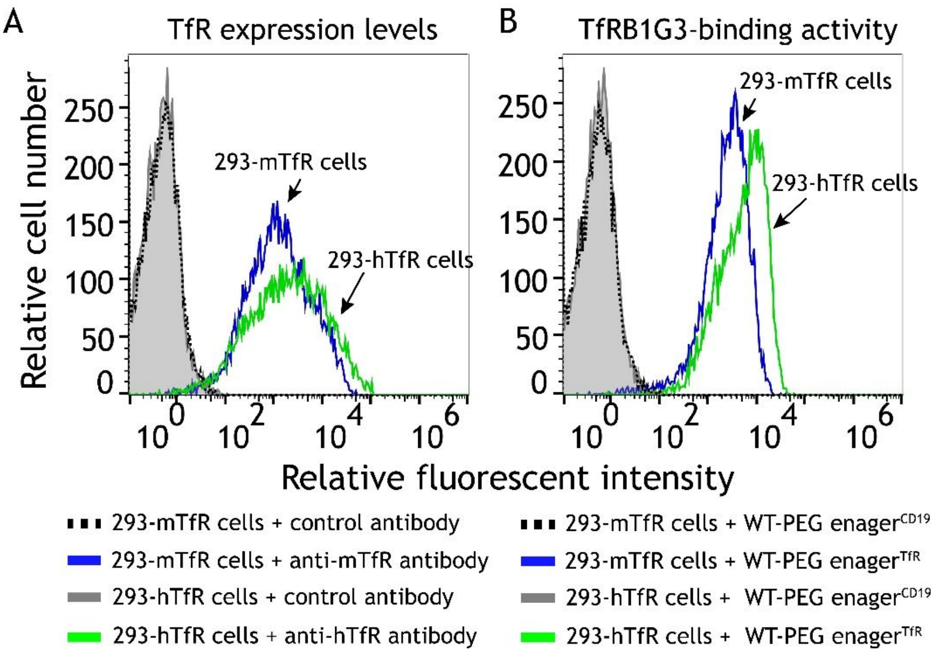

Figure S2. Cross-reactivity of TfRB1G3 against to mTfR and hTfR. (A) Live 293-mTfR or 293-hTfR cells were immunofluorescence stained with control, anti-mTfR, or anti-hTfR antibodies and then analyzed on a flow cytometer for determining the TfR expression levels in cells. (B) Live 293-mTfR or 293-hTfR cells were immunofluorescence stained with WT-PEG enager^{CD19} or WT-PEG enager^{TfR} antibodies and followed by PEG-lipoDiD staining. The cells were analyzed on a flow cytometer.

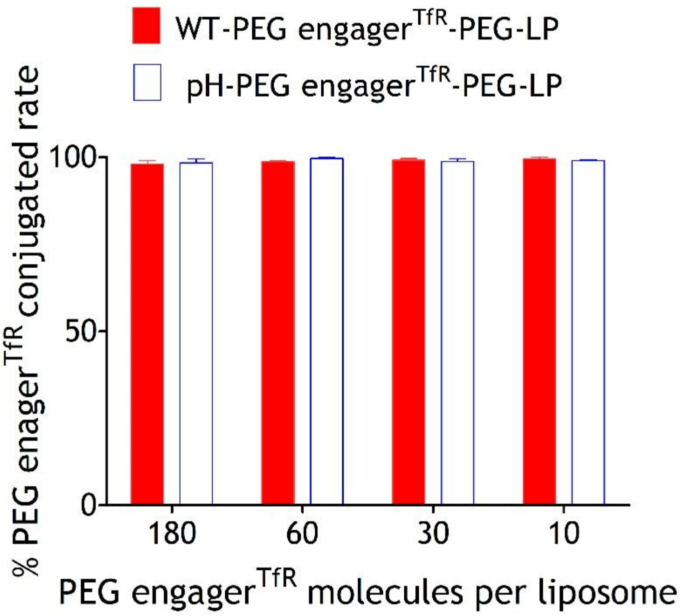

Figure S3. Coupling efficacy of PEG engager decorated PEG-LPs. The coupling rates of PEG engager decorated PEG-LPs were calculated according the formula: (total amount of PEG engagers – free PEG engagers) / total amount of PEG engagers × 100. Data are shown as mean ± standard deviation.

A

Engager/PEG-lipoDiO complex	Diameter (nm)	Zeta potential (mV)
PEG-lipoDiO	93.95 ± 0.63	-5.73 ± 0.90
30-DNS engager-PEG-lipoDiO	94.97 ± 0.36	-5.44 ± 1.26
30-WT-PEG engager- PEG-lipoDiO	103.01 ± 0.40	-11.03 ± 1.10
30-pH-PEG engager-PEG-lipoDiO	102.40 ± 1.31	-11.85 ± 1.25
60-DNS engager-PEG-lipoDiO	93.85 ± 0.35	-5.60 ± 0.45
60-WT-PEG engager-PEG-lipoDiO	103.35 ± 0.95	-11.50 ± 0.62
60-pH-PEG engager-PEG-lipoDiO	103.20 ± 0.60	-11.75 ± 1.55
180-DNS engager-PEG-lipoDiO	92.80 ± 0.66	-6.39 ± 2.39
180-WT-PEG engager-PEG-lipoDiO	121.10 ± 19.61	-15.05 ± 0.65
180-pH-PEG engager-PEG-lipoDiO	138.91 ± 31.70	-13.40 ± 1.12

B

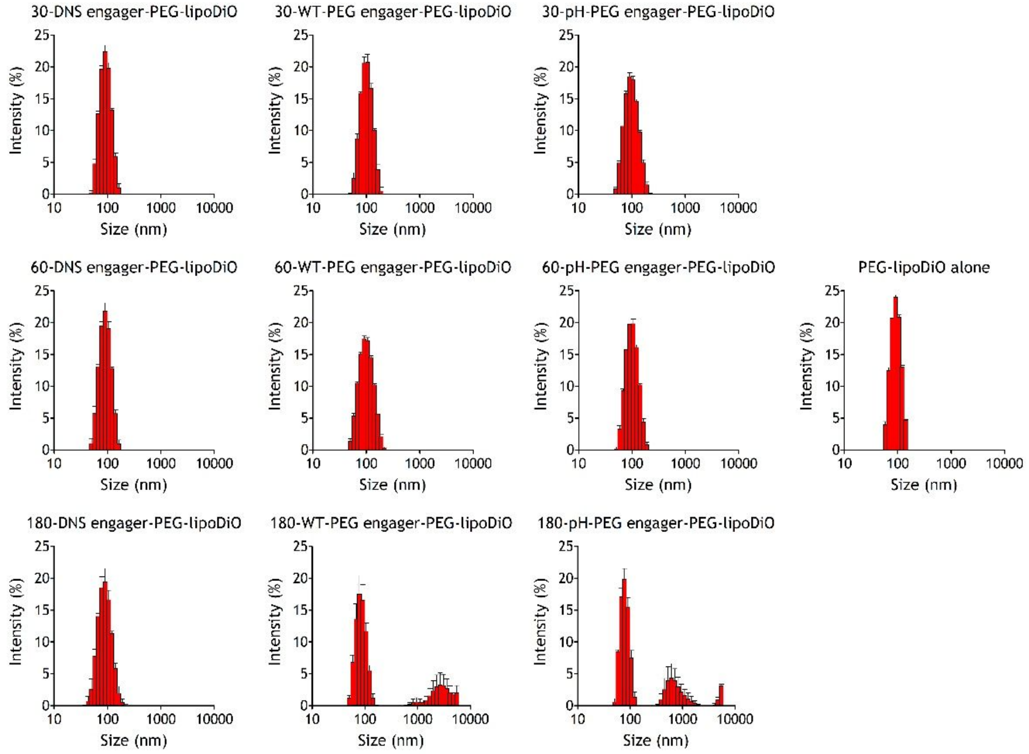

Figure S4. Physicochemical characterization of PEG engager decorated PEG-lipoDiO. (A) Average diameter and zeta potential of PEG-lipoDiO and DNS or PEG engager decorated PEG-lipoDiO. (B) Size distribution of PEG-lipoDiO and DNS or PEG engager decorated PEG-lipoDiO.

A

Engager/PEG-lipoDiD complex	Diameter (nm)	Zeta potential (mV)
PEG-lipoDiD	94.95 ± 0.92	-6.64 ± 0.36
30-DNS engager-PEG-lipoDiD	94.30 ± 1.05	-6.78 ± 0.90
30-WT-PEG engager- PEG-lipoDiD	103.50 ± 1.71	-13.50 ± 0.91
30-pH-PEG engager-PEG-lipoDiD	101.91 ± 1.82	-13.41 ± 0.32
60-DNS engager-PEG-lipoDiD	94.82 ± 0.15	-4.70 ± 1.51
60-WT-PEG engager-PEG-lipoDiD	102.71 ± 2.70	-13.03 ± 1.22
60-pH-PEG engager-PEG-lipoDiD	102.70 ± 3.39	-11.20 ± 2.10
180-DNS engager-PEG-lipoDiD	93.52 ± 1.29	-9.91 ± 0.40
180-WT-PEG engager-PEG-lipoDiD	147.35 ± 40.55	-14.92 ± 0.41
180-pH-PEG engager-PEG-lipoDiD	299.25 ± 29.15	-19.40 ± 0.62

B

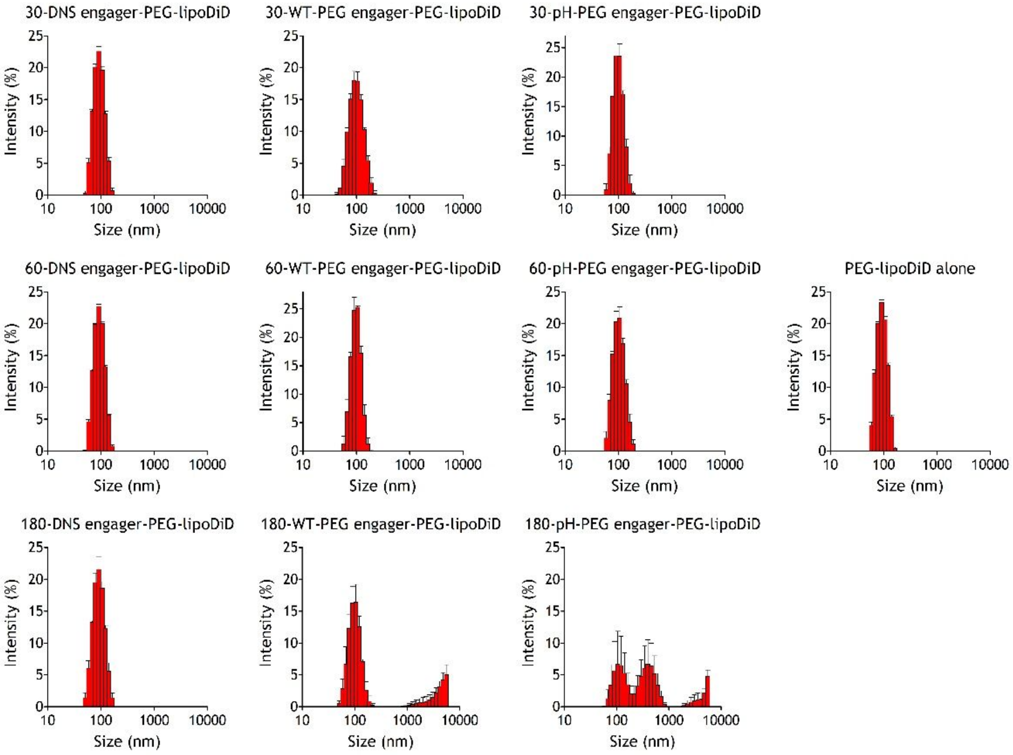

Figure S5. Physicochemical characterization of PEG engager decorated PEG-lipoDiD. (A) Average diameter and zeta potential of PEG-lipoDiD and DNS or PEG engager decorated PEG-lipoDiD. (B) Size distribution of PEG-lipoDiD and DNS or PEG engager decorated PEG-lipoDiD.

A

Engager/PEG-lipoDiR complex	Diameter (nm)	Zeta potential (mV)
PEG-lipoDiR	94.12 ± 0.33	-6.10 ± 0.33
30-DNS engager-PEG-lipoDiR	93.36 ± 0.65	-6.70 ± 0.78
30-WT-PEG engager- PEG-lipoDiR	97.54 ± 1.42	-13.30 ± 1.10
30-pH-PEG engager-PEG-lipoDiR	97.60 ± 0.64	-13.30 ± 0.30
60-DNS engager-PEG-lipoDiR	92.05 ± 0.81	-4.91 ± 1.80
60-WT-PEG engager-PEG-lipoDiR	98.88 ± 1.08	-13.10 ± 1.30
60-pH-PEG engager-PEG-lipoDiR	99.42 ± 2.95	-11.60 ± 2.50
180-DNS engager-PEG-lipoDiR	93.60 ± 0.80	-10.10 ± 0.50
180-WT-PEG engager-PEG-lipoDiR	148.30 ± 34.20	-15.10 ± 0.30
180-pH-PEG engager-PEG-lipoDiR	141.36 ± 34.36	-19.30 ± 0.70

B

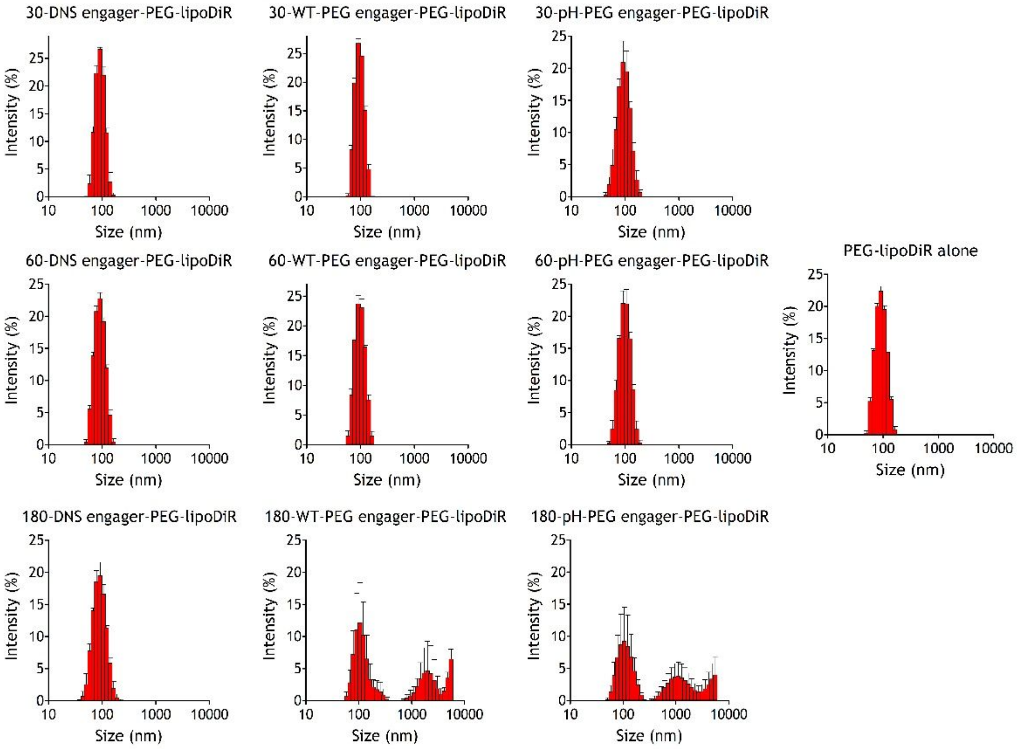

Figure S6. Physicochemical characterization of PEG engager decorated PEG-lipoDiR. (A) Average diameter and zeta potential of PEG-lipoDiR and DNS or PEG engager decorated PEG-lipoDiR. (B) Size distribution of PEG-lipoDiR and DNS or PEG engager decorated PEG-lipoDiR.

A

Engager/Doxisome complex	Diameter (nm)	Zeta potential (mV)
Doxisome	94.81 ± 0.60	-11.12 ± 1.70
30-DNS engager-Doxisome	94.10 ± 0.11	-10.59 ± 0.84
30-WT-PEG engager-Doxisome	101.60 ± 3.22	-16.30 ± 1.43
30-pH-PEG engager-Doxisome	101.51 ± 1.50	-15.90 ± 1.25
60-DNS engager-Doxisome	95.53 ± 1.51	-10.33 ± 0.84
60-WT-PEG engager-Doxisome	103.22 ± 2.63	-16.75 ± 1.13
60-pH-PEG engager-Doxisome	103.06 ± 3.02	-15.40 ± 0.65
180-DNS engager-Doxisome	95.20 ± 0.53	-9.69 ± 0.67
180-WT-PEG engager-Doxisome	253.41 ± 38.30	-18.37 ± 0.76
180-pH-PEG engager-Doxisome	225.30 ± 66.31	-18.19 ± 1.83

B

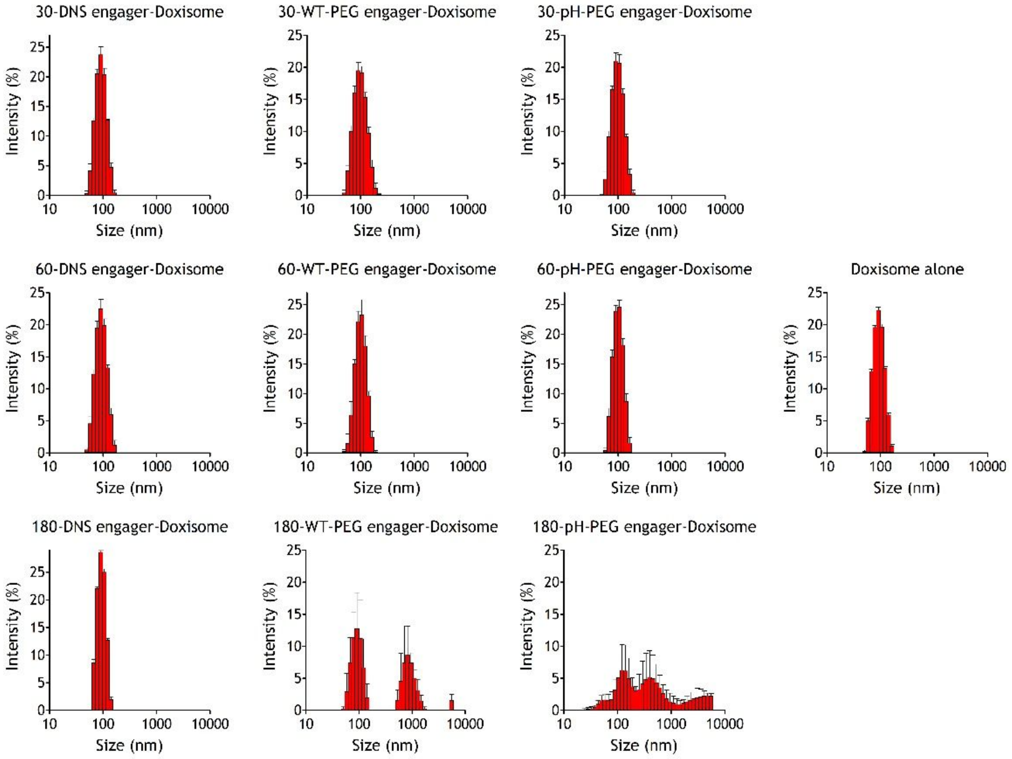

Figure S7. Physicochemical characterization of PEG engager decorated Doxisome. (A) Average diameter and zeta potential of Doxisome and DNS or PEG engager decorated Doxisome. (B) Size distribution of Doxisome and DNS or PEG engager decorated Doxisome.

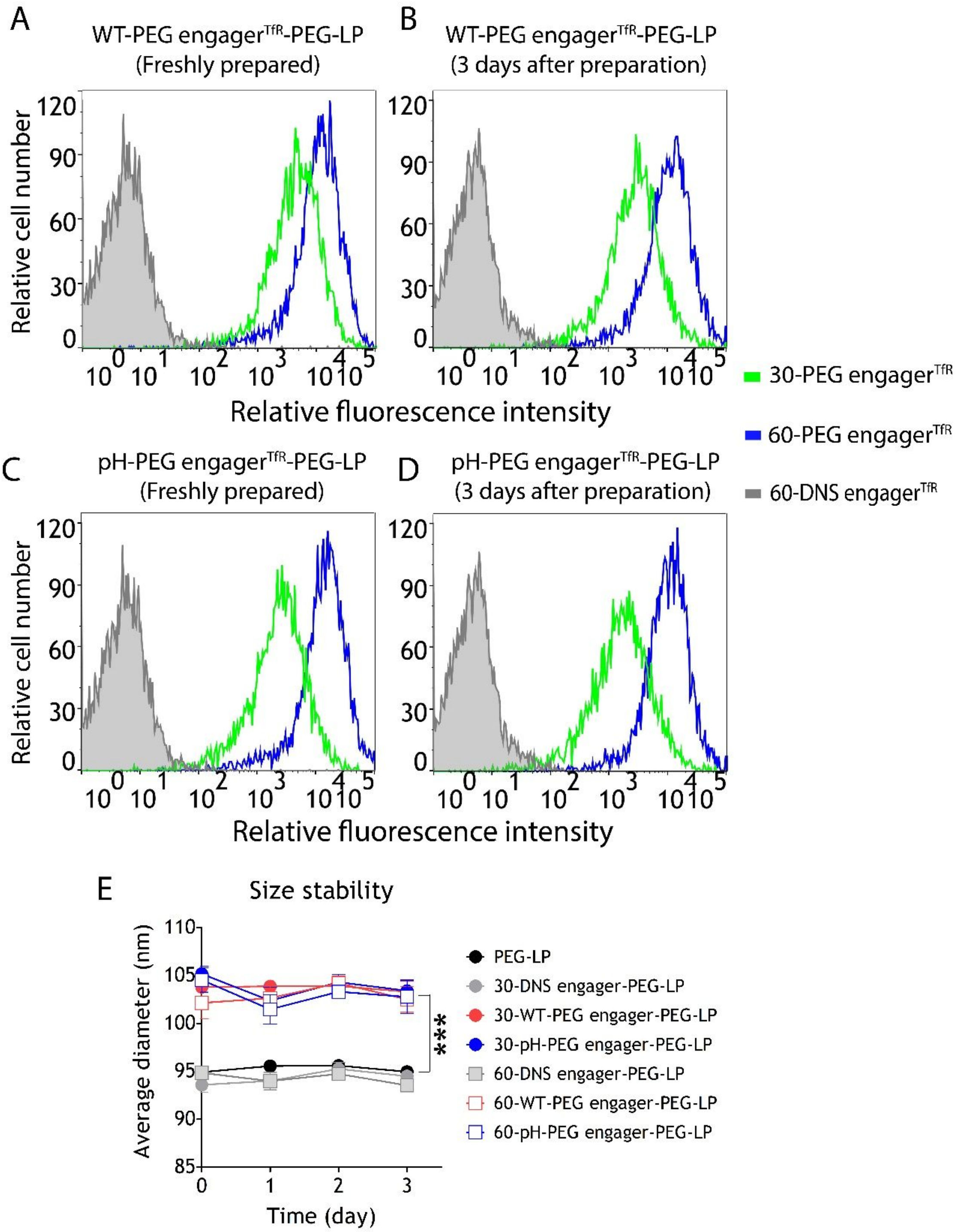

Figure S8. Stability of PEG engager decorated PEG-LPs. Various coupling ratios of PEG engager^{TfR} decorated fluorescent PEG-lipoDiD or control DNS engager^{TfR} decorated fluorescent PEG-lipoDiD prepared freshly (A and C) or for 3 days (B and D) were incubated with 293-mTfR cells followed by analyzed on a flow cytometer. (E) The size stability of PEG-LPs and PEG engager decorated PEG-LPs were assessed after being stored in buffer for 3 days. Two-way ANOVA was used for the statistical analysis. Data are shown as mean ± standard deviation. Significant differences in PEG-LP sizes between PEG engager decorated PEG-LPs and control groups are indicated: ***, $p \leq 0.001$.

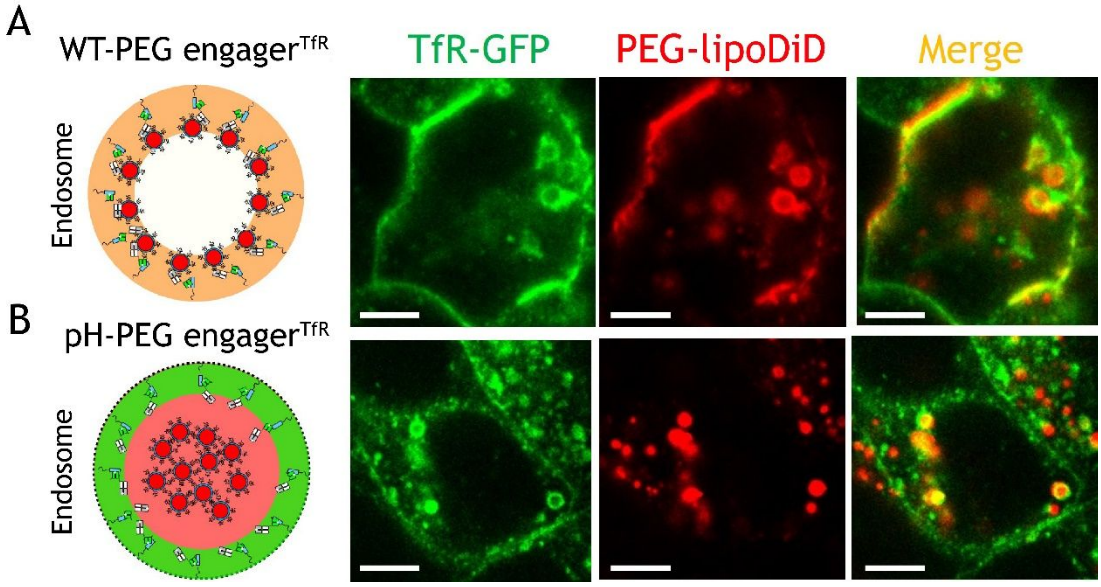

Figure S9. Analysis of co-localization of PEG engager decorated PEG-LPs with TfR-GFP in cells. Intracellular tracking of WT-PEG engager^{TfR} decorated PEG-lipoDiD (red) (A) or pH-PEG engager^{TfR} decorated PEG-lipoDiD (red) (B) in TfR-GFP overexpressing 293 cells (green). Scale bar, 10 µm.

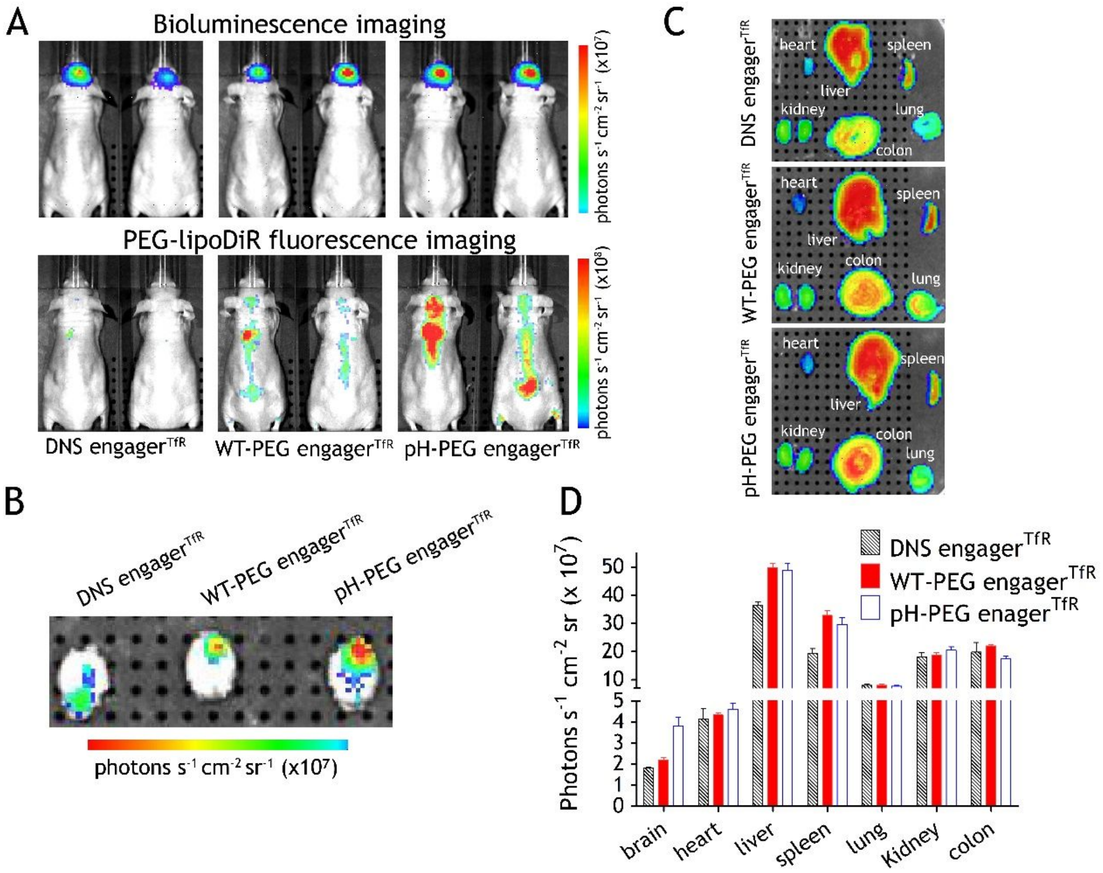

Figure S10. pH-PEG engager^{TfR} enhances brain uptake of PEG-NPs in GBM-bearing mice. (A) Bioluminescent and fluorescent images of intracranial U-87 MG-Luc2 GBM-bearing BALB/c nude mice that were intravenously injected with WT-PEG engager^{TfR}, pH-PEG engager^{TfR}, pH-PEG engager^{TfR}, or control DNS engager^{TfR} decorated PEG-lipoDiR. The whole-body imaging was imaged at 24 h with an IVIS® Spectrum imaging system (n = 2 mice). (B) *Ex vivo* fluorescence imaging of PBS-perfused tumor-bearing brains dissected from mice at 24 h post-injection. (C) *Ex vivo* fluorescence imaging of PBS-perfused organs isolated from mice at 24 h post-injection. (D) Quantitative biodistribution of PEG-lipoDiR fluorescence intensity in PBS-perfused organs collected from DNS or PEG engager decorated PEG-lipoDiR treated mice at 24 h post-injection. Data are shown as mean ± standard deviation.

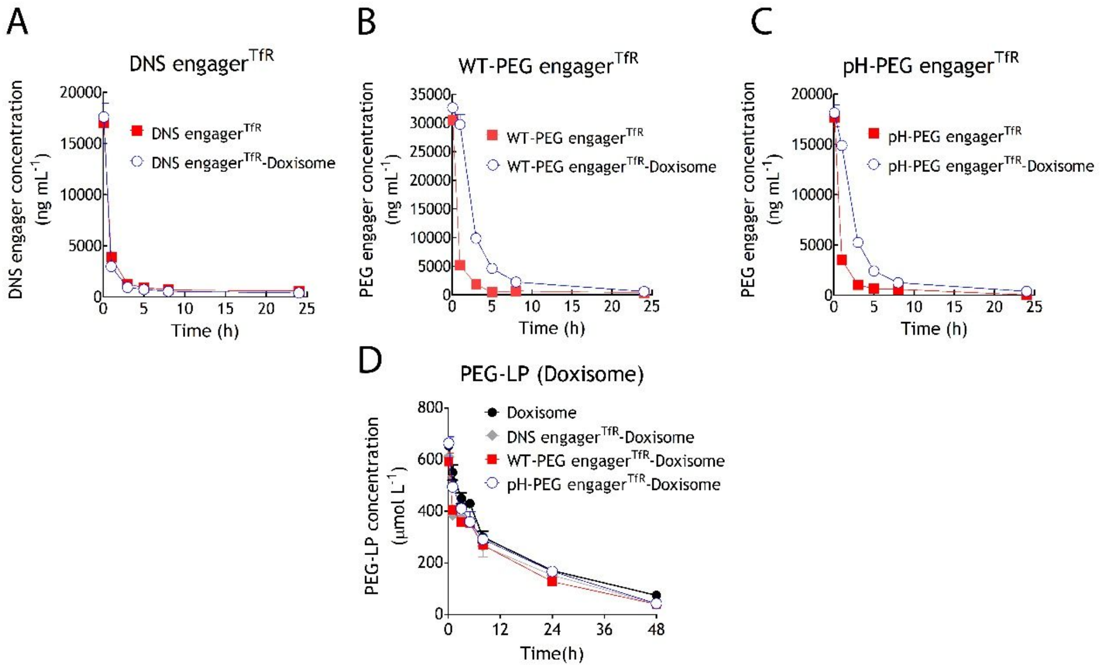

Figure S11. Pharmacokinetics of PEG engagers and PEG-LPs (Doxisome) in mice. BALB/c nude mice were intravenously injected with engager alone or engager decorated Doxisome (containing 45 μg of PEG engager). Mean plasma concentrations of the DNS (A), or PEG engagers (B and C), or Doxisomes (D) were measured by sandwich ELISAs.

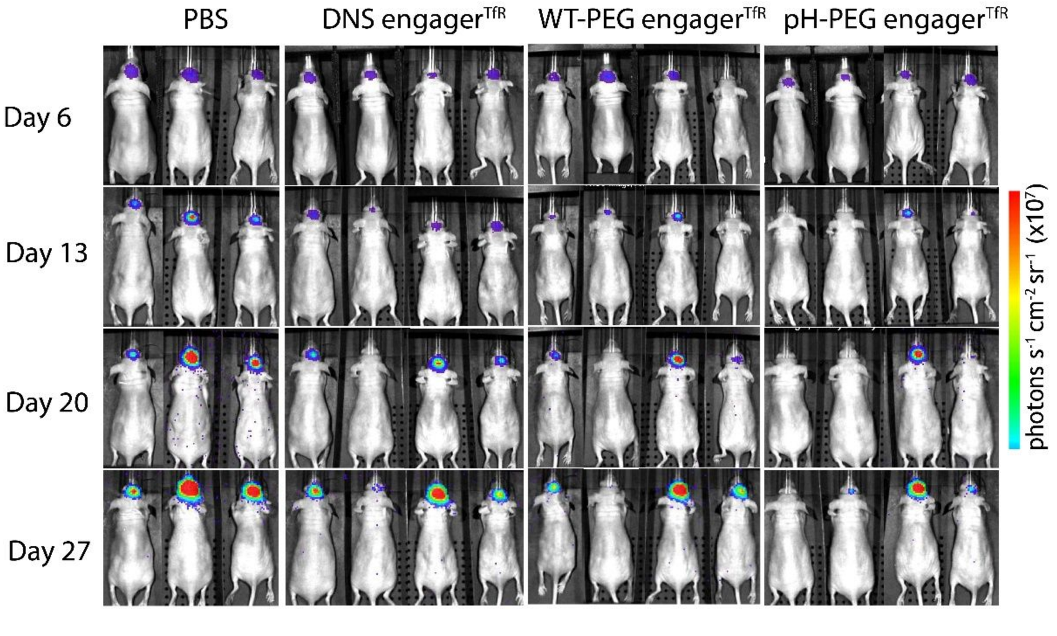

Figure S12. Anti-GBM efficacy of pH-PEG engager^{TfR} decorated Doxisome. DNS engager^{TfR}, WT-PEG engager^{TfR}, or pH-PEG engager^{TfR} was pre-mixed with Doxisome. Groups of six or eight BALB/c nude mice bearing intracranial U-87 MG-Luc2 GBM were intravenously injected with PBS alone, DNS engager^{TfR}, WT-PEG engager^{TfR}, or pH-PEG engager^{TfR} decorated Doxisome (3 mg kg^{-1}) once a week for 3 weeks. The bioluminescence corresponding to GBM growth was monitored by an IVIS Spectrum imaging system weekly. The representative bioluminescence images were shown.

Reference

(1) Huckaby, J. T.; Jacobs, T. M.; Li, Z.; Perna, R. J.; Wang, A.; Nicely, N. I.; Lai, S. K. Structure of an anti-PEG antibody reveals an open ring that captures highly flexible PEG polymers. *Commun. Chem.* **2020**, *3* (1), 124. DOI: 10.1038/s42004-020-00369-y.
